# The vascular targeted citrus *FLOWERING LOCUS T3* gene promotes non-inductive early flowering in transgenic Carrizo rootstocks and grafted juvenile scions

**DOI:** 10.1038/s41598-020-78417-9

**Published:** 2020-12-08

**Authors:** Juliana M. Soares, Kyle C. Weber, Wenming Qiu, Daniel Stanton, Lamiaa M. Mahmoud, Hao Wu, Patrick Huyck, Janice Zale, Kawther Al Jasim, Jude W. Grosser, Manjul Dutt

**Affiliations:** 1 Citrus Research and Education Center, University of Florida, Lake Alfred, FL 33850 USA; 2 Institute of Fruit and Tea, Hubei Academy of Agricultural Sciences, Wuhan, 430064 China; 3 Pomology Department, Faculty of Agriculture, Mansoura University, Mansoura, Egypt; 4 College of Agriculture, Al-Qasim Green University, Babylon, Iraq

**Keywords:** Plant biotechnology, Plant breeding, Plant molecular biology

## Abstract

Shortening the juvenile stage in citrus and inducing early flowering has been the focus of several citrus genetic improvement programs. FLOWERING LOCUS T (FT) is a small phloem-translocated protein that regulates precocious flowering. In this study, two populations of transgenic Carrizo citrange rootstocks expressing either *Citrus clementina FT1* or *FT3* genes under the control of the *Arabidopsis thaliana* phloem specific *SUCROSE SYNTHASE 2* (*AtSUC2*) promoter were developed. The transgenic plants were morphologically similar to the non-transgenic controls (non-transgenic Carrizo citrange), however, only *AtSUC2-CcFT3* was capable of inducing precocious flowers. The transgenic lines produced flowers 16 months after transformation and flower buds appeared 30–40 days on juvenile immature scions grafted onto transgenic rootstock. Gene expression analysis revealed that the expression of *SUPPRESSOR OF OVEREXPRESSION OF CONSTANS 1* (*SOC1*) and *APETALA1* (*AP1*) were enhanced in the transgenics. Transcriptome profiling of a selected transgenic line showed the induction of genes in different groups including: genes from the flowering induction pathway, *APETALA2/ETHYLENE RESPONSE FACTOR* (*AP2*/*ERF*) family genes, and jasmonic acid (JA) pathway genes. Altogether, our results suggested that ectopic expression of *CcFT3* in phloem tissues of Carrizo citrange triggered the expression of several genes to mediate early flowering.

## Introduction

In the genetic improvement of citrus by conventional breeding or biotechnology, the long juvenile phase is one of the major challenges encountered towards rapid evaluation of fruit quality^1^. Shortening this phase has been the focus of several studies^2–4^. In citrus, the transition from juvenility to maturity can range from as little as two years for Hong Kong kumquat, to over 12 years for some pummelo cultivars^2,5,6^. After this phase the plant transitions from the vegetative to the reproductive phase^7^. The process of flower development occurs in the shoot apical meristem (SAM), which transitions from producing vegetative structures to developing floral structures^8^.

**Figure 1:**
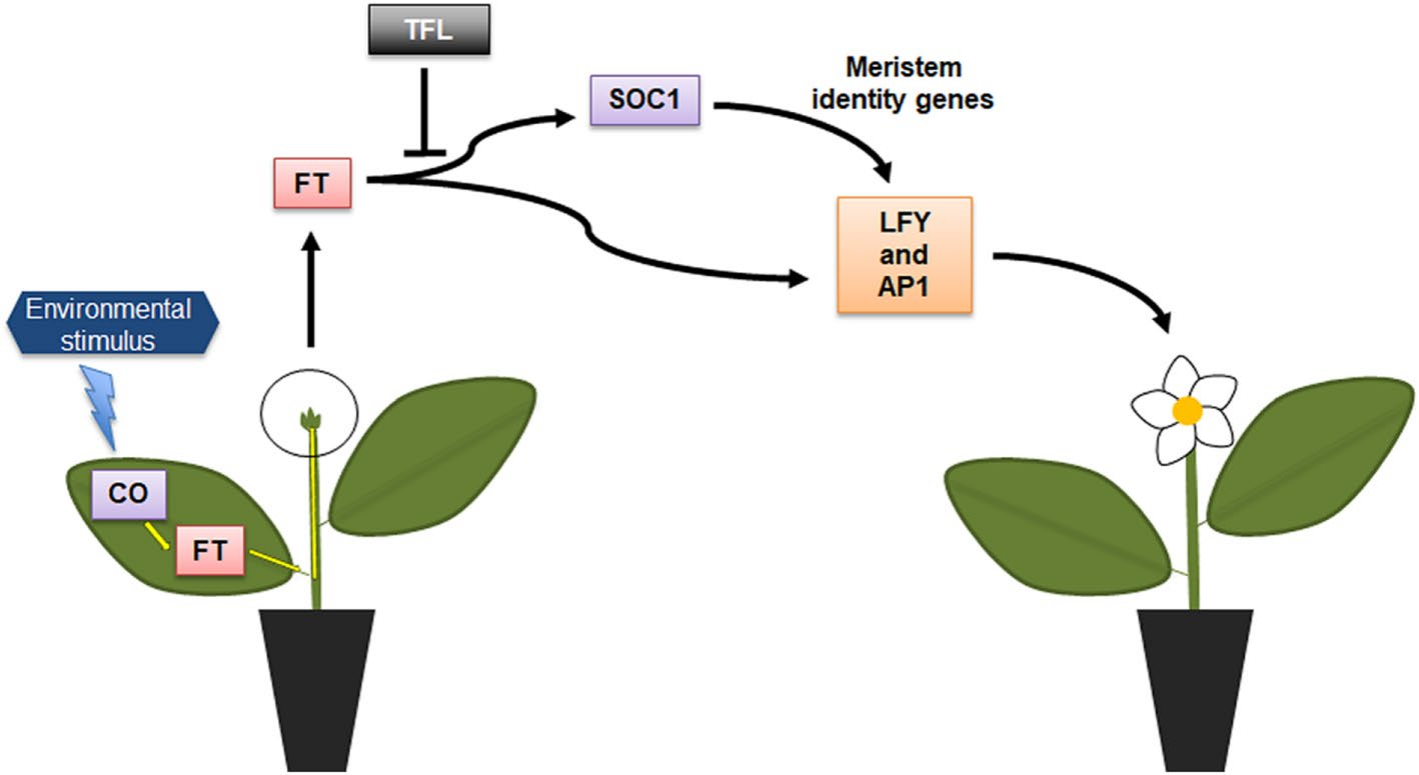
Schematic view of the flowering pathway in tropical and sub-tropical trees (adapted from Wilkie et al., 2008). Environmental stimuli promote the stabilization of the CO transcription factor, which upregulates FT protein. The FT protein is synthesized mainly in leaf tissue and transported by the phloem to the shoot apical meristem (SAM). In the SAM, FT induces flowering and TFL1 represses genes activated by FT. Downstream of FT, *SOC1* up-regulates floral meristem identity genes *AP1* and *LFY*. *FT* may also act independently of *SOC1* by inducing *AP1* and *LFY* gene expression. Up-regulation is represented by arrows and down-regulation or inhibition is represented by ‘an inverted T’**.**

Flowering is governed by a complex and finely tuned regulated network of signals. In *Arabidopsis*, four major regulatory pathways for flowering have been discovered including: the photoperiod pathway, autonomous floral initiation, the vernalization pathway, and those regulated by gibberellins. These pathways can be negatively or positively regulated by transcription factors, repressors, endogenous signal cascades, and/or environmental signals, which converge to regulate floral meristem identity genes that promote flowering^9–11^. *FLOWERING LOCUS T* (*FT*) is a key component of these flowering pathways. FT is a small protein synthesized in phloem companion cells which is upregulated by the transcription factor *CONSTANS* (*CO*). The FT protein translocates through the phloem to the SAM to induce floral primordia formation via the activation of meristem identity genes such as *APETALA1* (*AP1*)^12,13^ and *LEAFY* (*LFY*)^14,15^, converting the vegetative SAM into inflorescence SAM which later on starts producing flowers^10,16,17^ (Fig. 1).

Several studies have suggested that orthologs of the *A. thaliana* FT (AtFT) protein are a primary component of the flowering signal cascade in different plants^3,16,18–23^. When the tomato *FT*, also known as *SFT*, were overexpressed in either tomato or tobacco, an early flowering phenotype was observed. Subsequently, grafting experiments in tomatoes demonstrated that the signal was transmissible from transgenic plants expressing *SFT* under 35S promoter to non-transgenic *sft* mutant plants^24^.

In citrus, at least three loci encoding FT homolog proteins CiFT1, CiFT2 and CiFT3 have been identified^3,25^, however, two of them (CiFT1 and CiFT2) appear to be encoded by the same gene^26^. A shortened juvenile stage was observed when one of the homologs was ectopically expressed under the control of the *Cauliflower mosaic virus* (*CaMV 35S*) promoter in trifoliate orange (*Poncirus trifoliata* L. Raf.)^3^. In addition to the induction of early flowering phenotype, the overexpression of *CiFT* also induced abnormal morphological changes in the transgenic plants. Plants were dwarfed and their thorns replaced by flowers. This indicated that there were deleterious effects resulting from expression of *FT* gene under a strong constitutive promoter^3^. Similarly, the induction of flowering within four to six months was mediated by infection of citrus plants with a citrus leaf blotch virus-based vector expressing the FT protein (*CiFT* and *AtFT*)^4^. The virus infected plants showed no modification in plant, leaf, flower, or fruit morphology, suggesting that CiFT likely encodes a mobile floral signal and its expression does not lead to drastic phenotypic changes^4^.

In this study, we evaluated the early flowering phenotype using a *FT* citrus homolog cloned from *Citrus clementina* (*CcFT3)*. A transgenic population of Carrizo citrange rootstocks (*Citrus sinensis* Osb. × *Poncirus trifoliata* L. Raf.) transformed with the *AtSUC2-CcFT3* construct was generated*,* which showed normal morphological characteristics and exhibited normal vigor. Flowering occurred 16 months after transformation and clonally propagated transgenic rootstocks were able to induce precocious flowering in budded juvenile scions, demonstrating that the gene can induce earlier flowering in citrus.

## Results

### The *AtSUC2* promoter efficiently drives expression of the *CcFT3* transgene to induce early flowering in juvenile citrus

**Figure 2:**
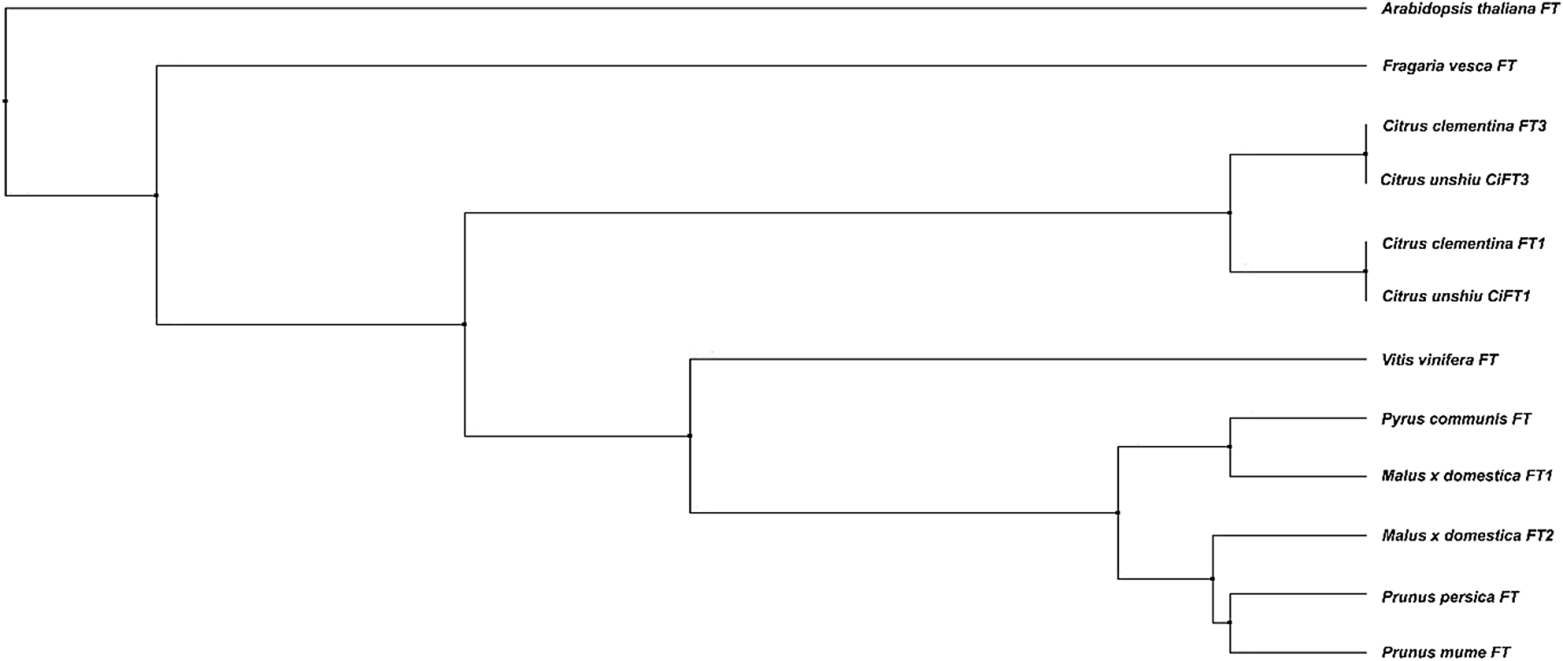
Phylogenetic analysis of FT protein sequences of multiple plant species. Sequences evaluated include: *Arabidopsis thaliana* FT (AAF03936), *Citrus clementina* FT1 (MT707614), *Citrus clementina* FT3 (MT602515), *Citrus unshiu* FT1 (BAA77836), *Citrus unshiu* FT3 (BAF96645), *Fragaria vesca* FT (CBY25183), *Malus domestica* FT1 (BAD08340),* Malus domestica* FT2 (ADP69290), *Prunus mume* FT (CBY25181),* Prunus persica* FT (AEO72030),* Pyrus communis* FT (AJC01933) and *Vitis vinifera* FT (ABF56526).

Three *FT* homologs have been identified in the satsuma mandarin (*Citrus unshiu*). Among the three, the *FT1* and *FT2* are considered alleles at the same locus^26,27^, and therefore, only *FT1* and *FT3,* was used in this study (Supplementary Fig. S1). In this study, we identified *FT1* and *FT3* from the clementine mandarin. Then, a phylogenetic tree was constructed based in the predicted CcFT protein sequences and compared them with several other FT sequences identified previously from the satsuma mandarin, other fruit crops and *Arabidopsis*. The phylogenetic tree was produced by the maximum-likelihood method. When compared to other methods such as the maximum-parsimony or the neighbor-joining, results were almost identical (data not presented). Both the *CcFT1* and *CcFT3* sequences were homologous to the *CiFT1* and *CiFT3*, were closely located and in the same clade, along with the CiFT3 (Fig. 2). The *CcFT1* and *CcFT3* cDNA sequences were cloned into binary vectors for *Agrobacterium-*mediated transformation of Carrizo citrange. We generated different populations of transgenic citrus plants for evaluation. Several promoters were examined to efficiently express the *CcFT* transgene. These included two constitutively expressed promoters (the strong *35S* promoter derived from *Cauliflower Mosaic Virus* (*CaMV*) and a weaker *NOPALINE SYNTHASE* (*NOS*) promoter), the *A. thaliana SUCROSE SYNTHASE 2* (*AtSUC2*) that localizes to vascular tissue^28^, and the *A. thaliana HEAT SHOCK PROTEIN 18.2* (*AtHSP18.2*) heat inducible promoter^29–32^ (Supplementary Fig. S2). Non-transformed Carrizo citrange rootstocks that originate from control in vitro plates were used as non-transgenic controls.

**Figure 3:**
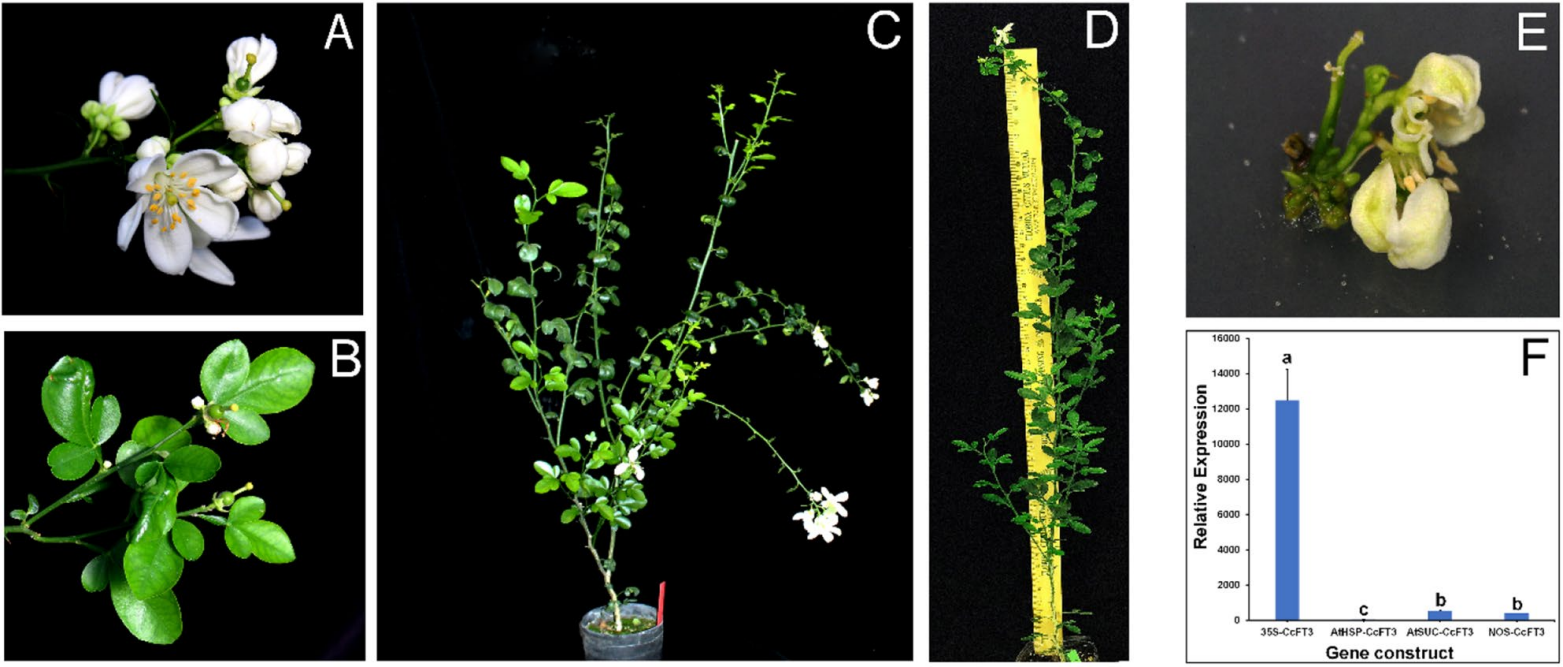
*AtSUC2-CcFT3* transgenic rootstock flowering phenotype. (**A**) Precocious flowering from a 16-month old transgenic *AtSUC2-CcFT3* plant. (**B**) Closeup of fruit set following self-pollination of the flowers. (**C**) A view of the whole plant. (**D**) Precocious flowering in a self-pollinated *AtSUC2-CcFT3* F1 seedling within a year of seed germination. (**E**) Precocious in vitro flowering within a month of transformation using a *35S*-*CcFT3* construct and (**F**) relative *CcFT3* gene expression of transgenic lines transformed with the different constructs. The error bars represent the means of different transgenic lines. Different letters represent a significant difference at P < 0.05 using Student's t-Test.

None of the transgenic *CcFT1* overexpressing lines flowered even after three years of transformation, heat treatment (to induce the *AtHSP18.2* promoter) and plant regeneration (Table 1). In contrast, *CcFT3* overexpression induced precocious flowering in many transgenic lines (Fig. 3A). Therefore, we focused on the evaluation of a *CcFT3* transgenic line.

**Table 1:** Summary of transgenic lines produced in this study.

Gene	Promoter	Function	Stable transgenic lines	Lines flowering
*CcFT1*	35S	Strong constitutive	31	0
	NOS	Weak constitutive	12	0
	AtHSP	Heat inducible	20	0
	AtSUC2	Phloem limited	35	0
*CcFT3*	35S	Strong constitutive	18	11*
	NOS	Weak constitutive	25	1**
	AtHSP	Heat inducible	30	0
	AtSUC2	Phloem limited	21	7

*These lines all flowered in vitro.

**Plant flowered once.

The transcripts levels were evaluated using CcFT3-specific primers to compare the expression of transgenic plants to the control plants (Supplementary Table S1). When the in vitro derived *CcFT3* transcript levels were compared, transgenic lines expressing the 35S-*CcFT3* construct (Fig. 3E) had on average over a 12,000-fold higher transgene expression than the *AtSUC2-CcFT3* transgenic lines (Fig. 3F). The expression of the transgene under the 35S promoter also resulted in precocious flowering in the apical meristems in vitro, however, the explant was unable to survive after soil transplantation (Fig. 3E).

One transgenic *NOS*-*CcFT3* line flowered within 18 months after transformation but did not flower in subsequent years. Forty lines expressing *CcFT3* under control of *AtHSP18.2* and 21 lines expressing *CcFT3* under control of *AtSUC2* promoter were regenerated and later transplanted to soil (Table 1). None of the *AtHSP18.2-CcFT3* plants flowered during the time span of this study.

**Figure 4:**
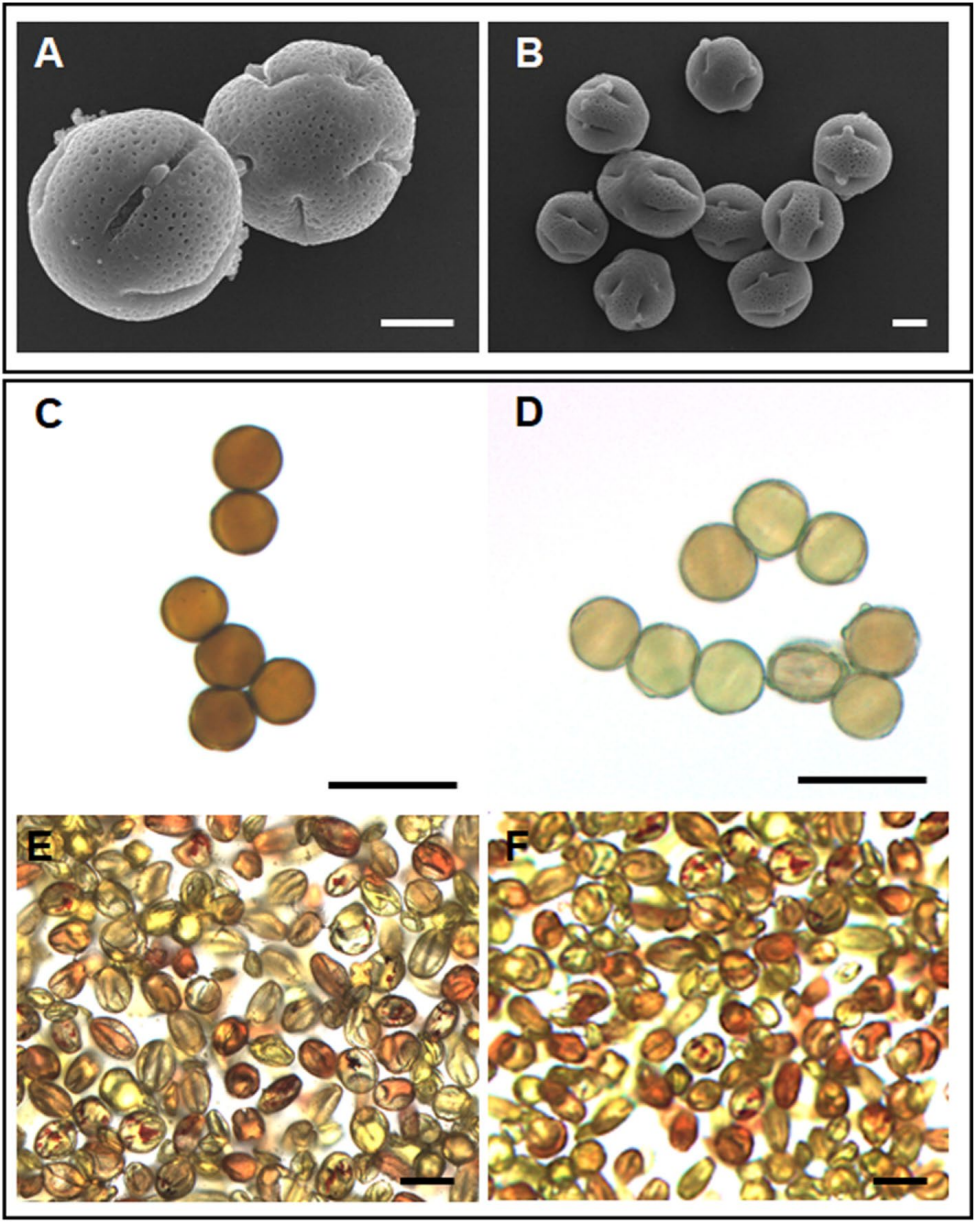
Pollen grains of a transgenic line showing normal structure and high viability. (**A**,**B**) SEM images of pollen grains from the *AtSUC2-CcFT3* MP3 transgenic line indicate normal morphology. (**B**) Brown–red pollen grains stained with 50% Gram’s iodine indicate that the pollen grains are viable compared to the (**C**) deionized water control. The 1% TTC stain showed that the relative number of viable pollen grains between the (**E**) MP3 transgenic line and the (**F**) non-transgenic line was the same, although differential staining was observed. Scale bars represent 10 µm.

Interestingly, seven out of 21 independent transgenic lines expressing *CcFT3* gene under the control of the *AtSUC2* promotor flowered within 16 months after transformation (Fig. 3A–C). Transgenic nucellar seedlings from fruit obtained from the *AtSUC2- CcFT3* lines flowered within a year after germination (Fig. 3D). The population was screened using *FT* and *nptII* specific primers and both *FT* and *nptII* gene fragments were successfully amplified from each of the transgenic lines (Supplementary Fig. S3). To distinguish between the native citrus *FT3* and our introduced construct, primers were designed to span a 700 bp fragment of the *AtSUC2* promotor and the *FT3* gene (Supplementary Table S1). The morphological characteristics of these *AtSUC2-CcFT3* transgenic trees were similar to the non-transgenic control. However, thorns in the transgenic lines were visibly smaller and were comparable to those usually seen in mature trees. There were no morphological abnormalities observed in the flowers in any of the transgenic lines (Fig. 3A) and a normal surface morphology of the pollen grains under SEM was observed (Fig. 4A,B). The number of viable pollen grains were similar between the transgenic line and the non-transgenic control (Fig. 4C–F). Controlled pollination between our transgenic lines and the monoembryonic citrus ‘Temple’ resulted in the production of viable hybrid seedlings with a trifoliate leaf phenotype (results not shown).

**Figure 5:**
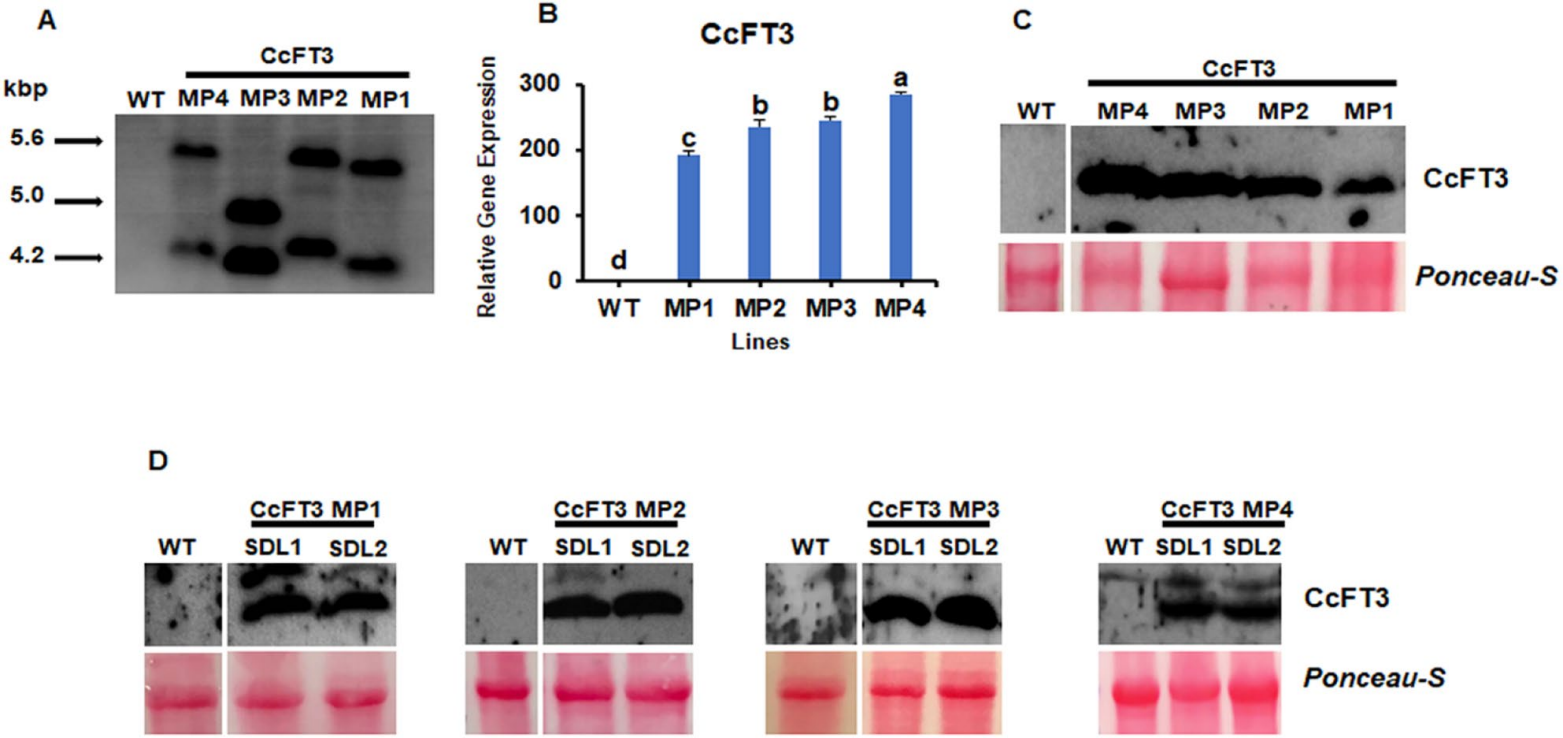
Molecular analysis of *Citrus* transgenic lines expressing *CcFT3* under control of the *AtSUC2* promoter. (**A**) Southern blot of selected *CcFT3* lines indicating the transgene copy number. The lanes were loaded as following: (1) WT or non-transgenic control, (2) AtSUC2-CcFT3 MP4, (3) AtSUC2-CcFT3 MP3, (4) AtSUC2-CcFT3 MP2 and (5) AtSUC2-CcFT3 MP1. (**B**) *CcFT3* transcript quantification from the selected citrus transgenic lines. *CsACTIN* was used as reference gene. Data represent the mean (± standard deviation, SD) of three technical replicates and different letters represent a significant difference at P < 0.05 using Student's t-Test (**C**–**F**) Western blot showing *CcFT3* protein quantification (**C**) in the mother plants (MP) and (**D**–**F**) two individual self-pollinated seedling progenies (SDL) from each MP line. The membranes were probed with CcFT3 specific antibody and *Ponceau-S* was used as loading control.

Among the seven PCR positive plants (Supplementary Fig. S3), four lines were randomly selected for further examination. The copy number of transgenes integrated in the plant genome of the transgenic lines was first evaluated by Southern blot hybridization. In all the transgenic plants, the presence of positive insertion of *CcFT3* transgene was observed compared to its absence in the control plant. Each of the transgenic lines contained two copies of the *CcFT3* transgene (Fig. 5A).

Transgenic lines were subsequently evaluated for *CcFT3* transcript levels. *CcFT3* could not be detected in the juvenile non-transgenic Carrizo citrange plants but was highly upregulated in the selected transgenic lines (Fig. 5B). In addition, the protein levels were correlated with PCR results (Fig. 5C). All the selected transgenic lines showed a high expression of the *CcFT3* transgene. The transgene stability was subsequently evaluated in their self-pollinated nucellar seedlings. Stable expression of the transgene in the nucellar seedlings was also detected in all the four lines (Fig. 5D). We also tested the ability of the *CcFT3* transgene to induce early flowering in a heterologous species*.* When the *CcFT3* transgene was inserted into *Arabidopsis* plants*,* either driven by the 35S or the *AtSUC2* promotor, early flowering was observed (Supplementary Fig. S4).

### Early flowering transgenic lines upregulate the floral transition process

**Figure 6:**
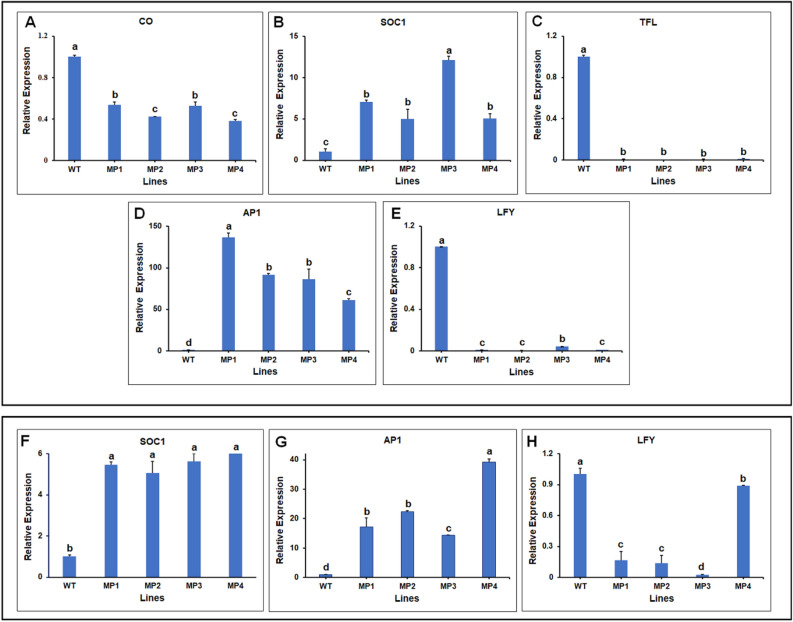
Evaluation of flowering pathway related genes. (**A**–**E**) Relative expression of flowering related genes for each of the 16-month old transgenic lines compared to the non-transgenic Carrizo citrange shown by RT-qPCR (**A**) *CONSTANS* (*CO*), (**B**) *SUPPRESSOR OF OVEREXPRESSION OF CONSTANS 1* (*SOC1*), (**C**) *TERMINAL FLOWER1* (*TFL1*), (**D**) *APETALA1* (*AP1*) and (E) *LEAFY* (*LFY*). RNA was extracted from leaf petioles rich in phloem tissues. (**F**–**H**) Total RNA was extracted from leaf tissues to show the expression flowering pathway genes by RT-qPCR (**A**) *SOC1*, (**B**) *AP1* and (**C**) *LFY* in the *AtSUC2-CcFT3* transgenic lines compared to the Carrizo citrange non-transgenic line. *CsACTIN* was used as the reference gene. Data represent the mean (± standard deviation, SD) of three technical replicates. Different letters represent a significant difference at P < 0.05 using Student's t-Test.

The effect of the transgene mediating an early flower phenotype in the *AtSUC2-CcFT3* transgenic lines by effecting gene expression in the flowering induction pathway was investigated. RT-qPCR was performed using RNA extracted from phloem-rich petioles. We did not observe a difference in the expression of *CONSTANS* (*CO*) between transgenic and non-transgenic plants (Fig. 6A). In contrast, *SUPPRESSOR OF OVEREXPRESSION OF CONSTANS 1* (*SOC1*) was induced and *TERMINAL FLOWER1* (*TFL1*) was repressed (Fig. 6B,C). The expression of the meristem-identity genes, *APETALA1* (*AP1*) and *LEAFY* (*LFY*) were examined. *AP1* was highly expressed in the transgenic lines, while *LFY* was repressed compared to non-transgenic plants (Fig. 6D,E). Similarly, genetic expression analysis from whole leaf showed the induction of *SOC1* and *AP1* but a reduced expression of *LFY* (Fig. 6F–H).

**Figure 7:**
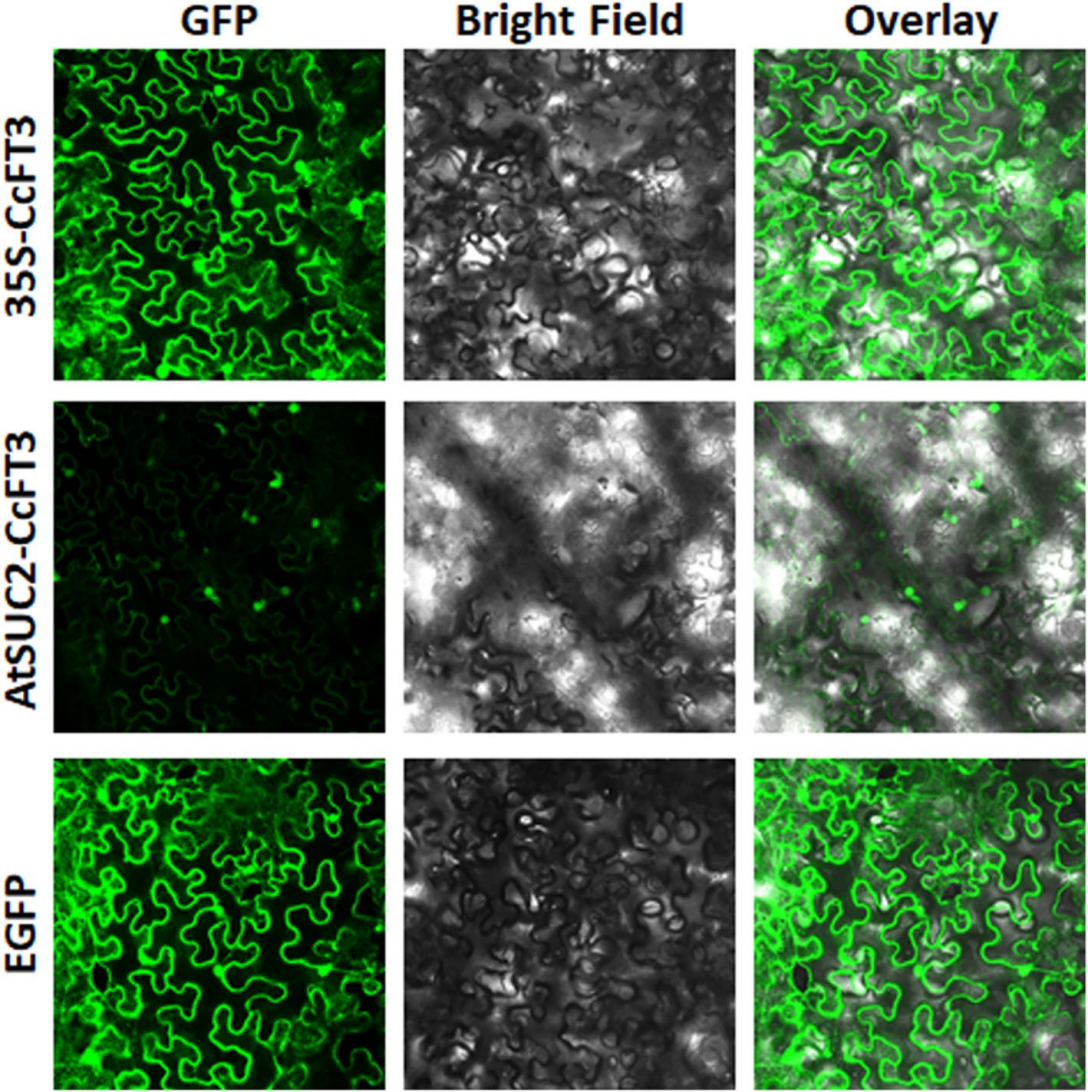
Confocal micrographs showing transient expression of the CcFT3-EGFP fusion protein. Confocal micrographs of GFP, transmission white light (Bright field) and an overlay. Cytoplasmic and nuclear subcellular localization of CcFT3 fused to EGFP under expression of both 35S and *AtSUC2* promoters was observed. EGFP was used as positive control.

To verify the transgene localization, *CcFT3-EGFP* fusions were generated. The fusion gene was cloned downstream of either the 35S or the *AtSUC2* promoters. Both constructs transiently expressed CcFT3-EGFP in *N. benthamiana* plants and exhibited cytoplasmic and nuclear localization (Fig. 7). When the fusion gene was driven by the 35S, a stronger EGFP signal was observed indicating higher protein expression when compared to the EGFP signal observed from the same fusion gene under the control of the weaker phloem specific promoter, *AtSUC2*.

### Transgenic rootstocks induced earlier flowering in non-transgenic juvenile scions

**Figure 8:**
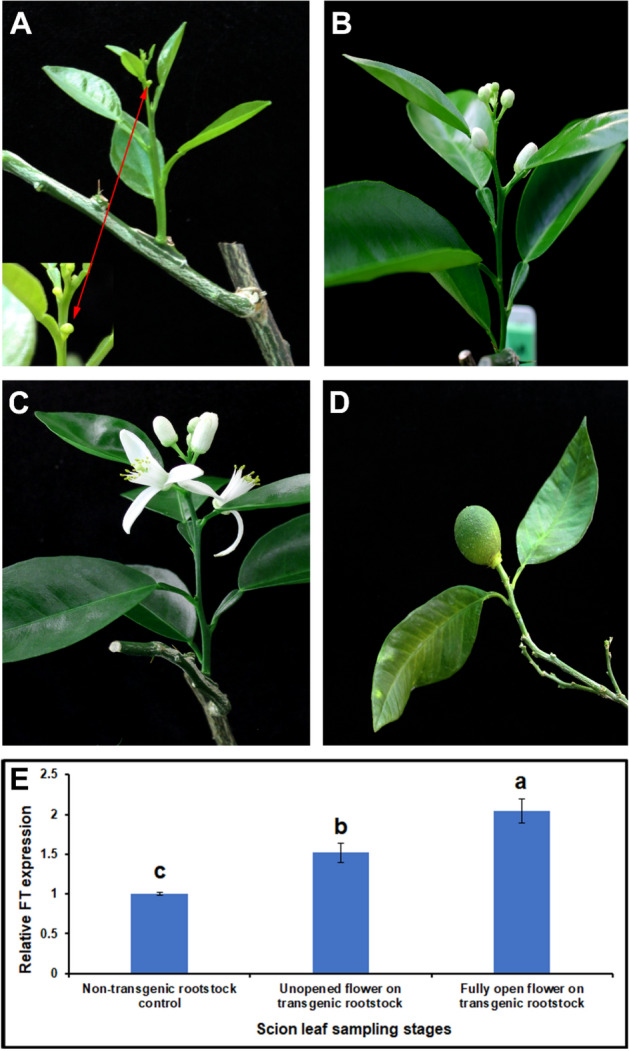
Precocious flowering one-year old non-transgenic ‘Valencia’ scion grafted onto *AtSUC2-CcFT3* transgenic rootstock (**A**) Flower bud emergence within 21 days following budding. Insert shows enlarged image of emerging flower buds (**B**) fully expanded flower buds, (**C**) fully open flowers, (**D**) developing sweet orange fruit and (**E**) relative *FT* gene expression in the scion leaves at different stages of flowering. Data represent the mean (± standard deviation, SD) of three technical replicates. Different letters represent a significant difference at P < 0.05 using Student's t-Test.

To understand the ability of the transgenic rootstocks to efficiently induce flowering in non-transgenic scions, the MP3 transgenic line was propagated using two node cuttings. Most of the cuttings were capable of rooting, producing morphologically normal plants. Flower bud initiation was observed within 21 days after budding when buds from a 1-year old juvenile ‘Valencia’ seedling were grafted onto pencil-diameter transgenic Carrizo rootstock lines (Fig. 8A). The flowers developed normally (Fig. 8B) and fully open flowers were observed within 10 days from bud initiation (Fig. 8C). When allowed to self-pollinate, fruit formation was observed (Fig. 8D). FT transcript levels increased progressively in the developing scion and was two-fold higher compared to shoots emerging from the non-transgenic rootstocks at the fully open flower stage (Fig. 8E).

### Expression of *CcFT3* gene in the phloem tissues alters the plant’s transcriptome

To reveal the differences in transcript profiles between the transgenic and non-transgenic plants, a transcriptome analysis was performed. The total RNA of three technical replicates of leaf samples from non-transgenic and the *AtSUC2-CcFT3* MP3 transgenic plants was subject to next-generation sequencing (RNAseq). The Illumina NovaSeq platform produced on average 22,070,506 and 22,492,946 raw read counts for the Carrizo citrange non-transgenic plants and *AtSUC2-CcFT3* (FT3) transgenic lines, respectively (Supplementary Table S2).

**Figure 9:**
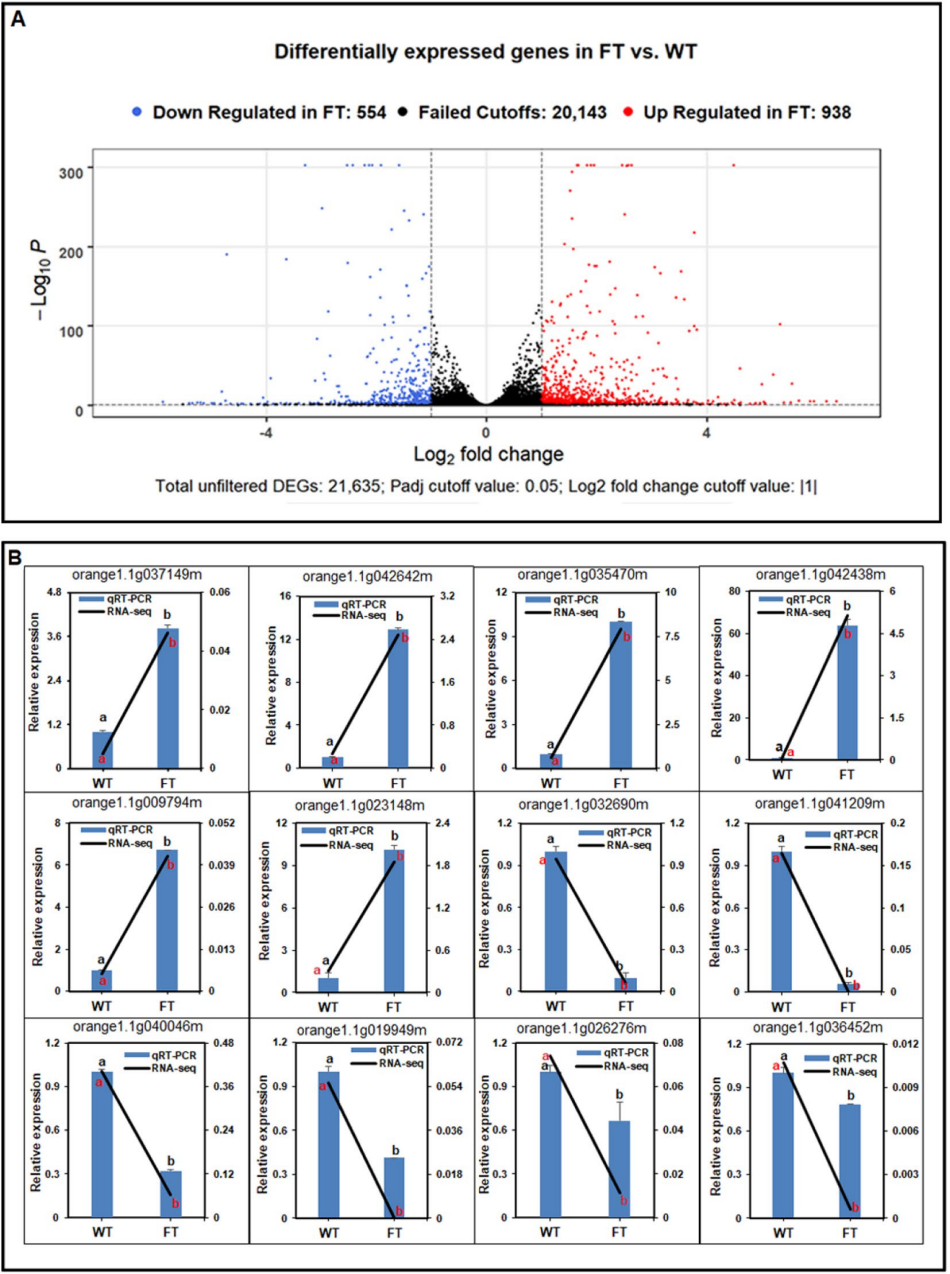
A volcano plot of DEGs and validation of the transcriptome analysis of selected DEGs using RT-qPCR. (**A**) Volcano plot showing the Log_2_ fold change difference and the adjusted P-value for 21,635 genes included on all platforms. The red dots represent upregulated genes and the blue dots the downregulated genes in *AtSUC2-CcFT3* MP3 transgenic lines (adjusted P < 0.05). (**B**) The expression levels of DEG candidates in *AtSUC2-CcFT3* MP3 transgenic line was calculated using 2^-ΔΔCt^ and those values were compared to non-transgenic control values. Different letters (a, b) represent a significant difference at P ≤ 0.05 using Duncan’s Multiple Range Test and error bars represent SE (n = 3) for RT-qPCR (black) and RNAseq (blue).

After cleaning, an average of 20,980,971 (95.06%) and 21,465,005 (95.43%) read counts remained. The clean reads were mapped to the *C. sinensis* genome utilizing STAR (version v2.6.0C, https://github.com/alexdobin/STAR) and the *C. sinensis* 154 v1.1 annotation and genome from Phytozome (version v12.1, https://phytozome.jgi.doe.gov/pz/portal.html). On average there were 17,272,422 and 17,478,222 unique alignments per sample, which accounted for a percent of 82.32% and 81.43%, of the genome respectively (Supplementary Table S2). A principal component analysis (PCA) in two dimensions was performed to demonstrate the variance between the samples of the transgenic and non-transgenic groups (Supplementary Fig. S5). A total of 1492 differentially expressed genes (DEGs) remained from the filtering of DESeq2 software analysis with a |log^2FoldChange^|≥ 1 and an adjusted P-value ≤ 0.05, of which 938 were upregulated and 554 were downregulated in the MP3 transgenic line when compared to the non-transgenic line (Fig. 9A).

To evaluate the accuracy of the RNAseq data and the presence of technical artifacts or errors introduced during the RNAseq library preparations, the expression of several well conserved genes and transcription factors were assayed through RT-qPCR. Six of the selected genes, including orange1.1g042438m (*CsAGL14*), orange1.1g035470m (*CsAGL8*), orange1.1g042642m (*CsAP1*), orange1.1g023148m (*CsERF38*), orange1.1g037149m (*CsAGL6*) and orange1.1g009794m (*CsWRKY61*), were highly upregulated, while orange1.1g041209m (*CsHSP70*), orange1.1g032690m (*CsWRKY23*), orange1.1g040046m (*CsAP3*), orange1.1g019949m (*CsGA2OX8*), orange1.1g036452m (*CsAGL11*) and orange1.1g026276m (*CsAP2*) were downregulated. The RT-qPCR results were consistent with the RNAseq data and the mRNA expression of these genes were significantly either up- or down-regulated by the transgenic expression of *CcFT3* (Fig. 9B).

**Figure 10:**
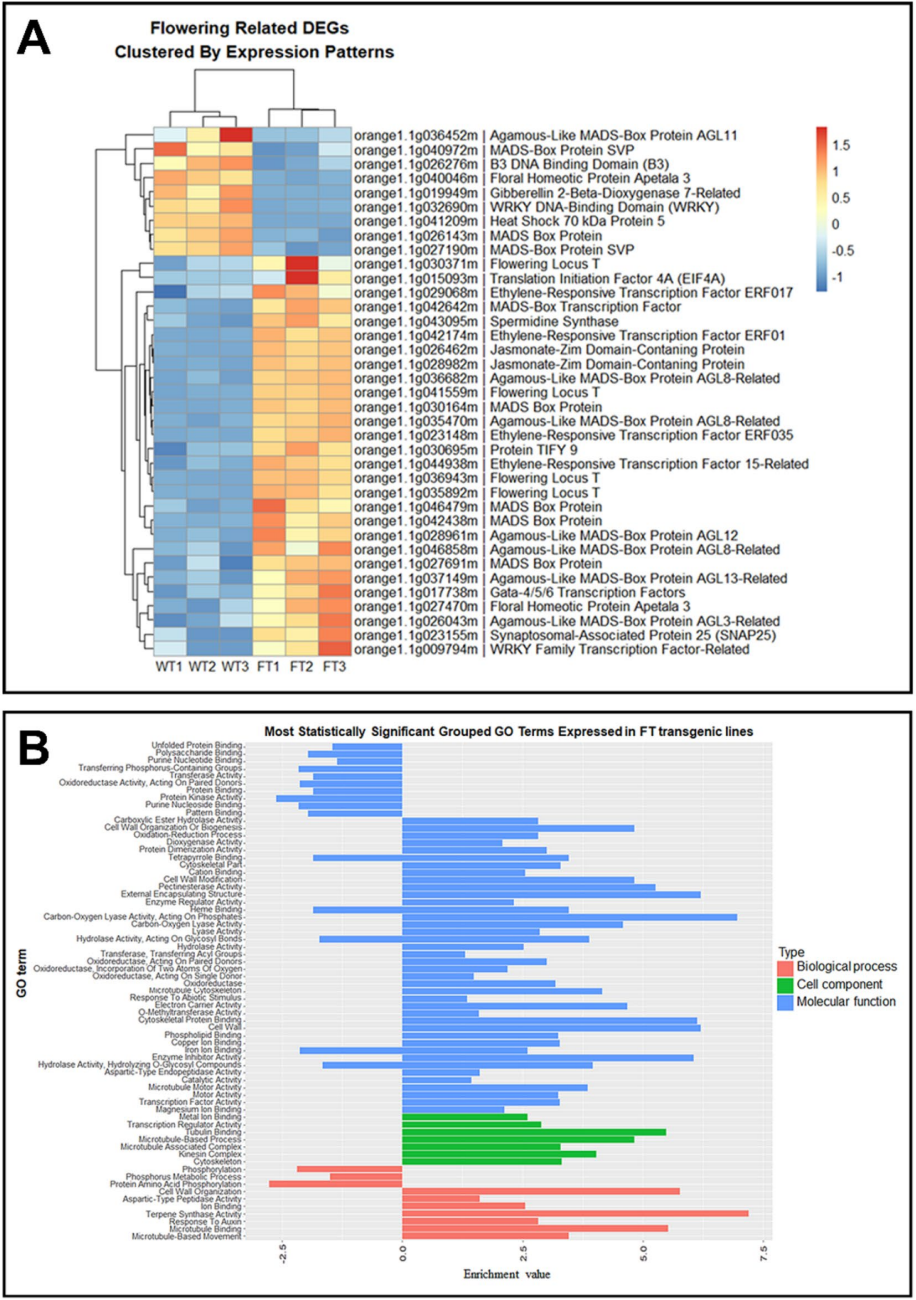
Heat map of RNAseq transcriptome analysis of 50 selected genes clustered by expression patterns and Gene Ontology (GO) enrichment analysis. **(A**) Heatmap displaying changes in gene expression between the MP3 transgenic line and non-transgenic plants. The DEGs were classified in flowering genes, *APETALA2/ETHYLENE RESPONSE FACTOR* (*AP2*/*ERF*) family, the jasmonic acid (JA) pathway. A color range of dark to light red represents upregulated genes in the transgenic lines with the most upregulated genes being dark red. A color range of dark to light blue represents the downregulated genes in the transgenic line with dark blue being the most downregulated genes (**B**) Bar graph showing the enrichment of gene ontology terms in differentially expressed genes in the MP3 transgenic line. In the x-axis the enrichment values are represented and GO terms are represented in the y-axis. The blue bars represent biological processes, green bars represent cellular components, and the red bars represent molecular function.

Among the 1492 DEGs, 50 genes which are relevant to flowering time and were either up- or down-regulated were selected to be included in a heatmap. These DEGs were further classified into different groups including flowering induction pathway genes, the *APETALA2/ETHYLENE RESPONSE FACTOR* (*AP2*/*ERF*) family of gene and the jasmonic acid (JA) pathway genes (Fig. 10A and Table 2).

**Table 2:** Flowering pathway related up- or down-regulated DEGs.

Phytozome accession no	Phytozome name	Log^2FoldChange^	*Arabidopsis* accession no	TAIR name
orange1.1g036943m	*FLOWERING LOCUS T2-1*	9.212929767	AT1G65480	*FLOWERING LOCUS T2-1*
orange1.1g035892m	*FLOWERING LOCUS T1*	7.198300851	AT1G65480	*FLOWERING LOCUS T1*
orange1.1g041559m	*FLOWERING LOCUS T2-2*	4.610520904	AT1G65480	*FLOWERING LOCUS T2-2*
orange1.1g030371m	*FLOWERING LOCUS T3*	2.492955066	AT1G65480	*FLOWERING LOCUS T3*
orange1.1g042438m	*MADS BOX PROTEIN*	6.576232451		*AGAMOUS-LIKE 14*
orange1.1g030164m	*MADS BOX PROTEIN*	3.778132987	AT5G60910	*AGAMOUS-LIKE 8–1*
orange1.1g035470m	*AGAMOUS-LIKE MADS-BOX PROTEIN AGL8-RELATED*	3.690178076	AT5G60910	*AGAMOUS-LIKE 8–2*
orange1.1g046858m	*AGAMOUS-LIKE MADS-BOX PROTEIN AGL8-RELATED*	1.975284628	AT5G60910	*AGAMOUS-LIKE 8–3*
orange1.1g037149m	*AGAMOUS-LIKE MADS-BOX PROTEIN AGL13-RELATED*	3.27128492	AT2G45650	*AGAMOUS-LIKE 6–1*
orange1.1g046479m	*MADS BOX PROTEIN*	2.572173653	AT2G45650	*AGAMOUS-LIKE 6–2*
orange1.1g036682m	*AGAMOUS-LIKE MADS-BOX PROTEIN AGL8-RELATED*	3.27270231	AT1G69120	*APETALA 1–1*
orange1.1g042642m	*MADS-BOX* transcription factor	3.212074085	AT1G69120	*APETALA 1–2*
orange1.1g027470m	*FLORAL HOMEOTIC PROTEIN APETALA 3*	1.507135173	AT3G54340	*APETALA 3*
orange1.1g017738m	*GATA-4/5/6* transcription factors	1.155652316	AT3G02380	*CONSTANS-LIKE 2*
orange1.1g028961m	*AGAMOUS-LIKE MADS-BOX PROTEIN AGL12*	1.122262674	AT1G71692	*AGAMOUS-LIKE 12*
orange1.1g027691m	*MADS BOX PROTEIN*	1.049289184	AT2G45660	*AGAMOUS-LIKE 20*
orange1.1g026143m	*MADS BOX PROTEIN*	-1.995966965	AT3G02310	*SEPALLATA 2*
orange1.1g026043m	*AGAMOUS-LIKE MADS-BOX PROTEIN AGL3-RELATED*	1.080045725	AT3G02310	*SEPALLATA 2*
orange1.1g036452m	*AGAMOUS-LIKE MADS-BOX PROTEIN AGL11*	-4.030135267	AT4G18960	*AGAMOUS*
orange1.1g026276m	*B3 DNA* binding domain (B3)	-2.67328835	AT5G42700	*APETALA 2*
orange1.1g040046m	*FLORAL HOMEOTIC PROTEIN APETALA 3*	-2.627403916	AT3G54340	*APETALA 3*
orange1.1g040972m	*MADS-BOX PROTEIN SVP*	-1.751862242	AT2G22540	*AGAMOUS-LIKE 22–1*
orange1.1g027190m	*MADS-BOX PROTEIN SVP*	-1.34053143	AT2G22540	*AGAMOUS-LIKE 22–2*
orange1.1g023148m	*ETHYLENE-RESPONSIVE TRANSCRIPTION FACTOR ERF035*	2.624954534	AT2G35700	*ETHYLENE RESPONSIVE FACTOR 38*
orange1.1g042174m	*ETHYLENE-RESPONSIVE TRANSCRIPTION FACTOR ERF01*	2.421205717	AT3G23240	*ETHYLENE RESPONSIVE FACTOR 1*
orange1.1g029068m	*ETHYLENE-RESPONSIVE TRANSCRIPTION FACTOR ERF017*	1.438029469	AT1G19210	*ETHYLENE RESPONSIVE FACTOR 17*
orange1.1g044938m	*ETHYLENE-RESPONSIVE TRANSCRIPTION FACTOR 15-RELATED*	1.71562111	AT4G17500	*ETHYLENE RESPONSIVE ELEMENT BINDING FACTOR 1*
orange1.1g026462m	*JASMONATE-ZIM DOMAIN-CONTANING PROTEIN*	3.771429514	AT1G19180	*JASMONATE-ZIM-DOMAIN PROTEIN 1–1*
orange1.1g028982m	*JASMONATE-ZIM DOMAIN-CONTANING PROTEIN*	2.735972905	AT1G19180	*JASMONATE-ZIM-DOMAIN PROTEIN 1–2*
orange1.1g030695m	*PROTEIN TIFY 9*	1.804727622	AT5G13220	*JASMONATE-ZIM-DOMAIN PROTEIN 10*
orange1.1g015093m	*TRANSLATION INITIATION FACTOR 4A (EIF4A)*	4.592345088	AT3G13920	*EUKARYOTIC TRANSLATION INITIATION FACTOR 4A1*
orange1.1g023155m	*SYNAPTOSOMAL-ASSOCIATED PROTEIN 25 (SNAP25)*	3.281267505	AT1G13890	*NSF ATTACHMENT PROTEINS RECEPTOR*
orange1.1g043095m	*SPERMIDINE SYNTHASE*	3.131463403	AT1G70310	*SPERMIDINE SYNTHASE 2*
orange1.1g009794m	*WRKY FAMILY TRANSCRIPTION FACTOR-RELATED*	2.971657574	AT5G15130	*WRKY 72* transcription factor

There were 23 genes identified and included in the flowering genes group, as they belong to an integrated network of several genetic pathways converging on flowering. An ortholog of the *Arabidopsis AGL14*, orange1.1g042438m and two *AGL6* like genes, annotated in the sweet orange genome database as orange1.1g037149m and orange1.1g046479m, were upregulated in our MP3 transgenic line. Further, two sweet orange genes (orange1.1g042642m and orange1.1g027470m) identified as the *APETALA* gene *AP1* and *AP3*, were upregulated. In contrast, a second *AP3* orthologue, orange1.1g040046m, was down regulated (Fig. 10A).

The *APETALA2/ETHYLENE RESPONSE FACTOR* (*AP2*/*ERF*) gene family is a group of plant-specific TFs that contain at least one *AP2* binding domain. Two sweet orange genes (orange1.1g023148m and orange1.1g029068m) were identified as orthologs of the *EARLIER DEHYDRATION RESPONSIVE ELEMENT BINDING PROTEINS* (*DREB*) and two sweet orange orthologs (orange1.1g042174m and orange1.1g044938m) were identified as orthologs of the *ETHYLENE RESPONSE FACTOR* (*ERF*) (Fig. 10A).

There were three orange orthologs of the JA pathway genes. Two of them were identified as *JASMONATE-ZIM DOMAIN1* (*JAZ1*), orange1.1g026462m and orange1.1g028982m, and one of them as *JAZ10*, orange1.1g030695m. Finally, other upregulated genes in the MP3 transgenic associated with the induction of early flowering were orange orthologs of *WRKY72* (orange1.1g009794m), *TRANSLATION INITIATION FACTOR 4A* (*EIF4A*) (orange1.1g015093m)*, SYNAPTOSOMAL-ASSOCIATED PROTEIN 25* (*SNAP25*) (orange1.1g023155m), and *SPERMIDINE SYNTHASE* (orange1.1g043095m) (Fig. 10A)^33–36^.

Using *C. sinensis* genome information, gene ontology (GO) enrichment analysis was performed to extract biological meaning of the DEGs obtained from the transcriptome. The *C. sinensis* genome annotation is composed of 25,380 genes from which 13,309 (52.44%) genes have GO terms. In addition, the total of filtered DEGs found in our RNAseq analysis were 1492 from which 847 (56.77%) have GO terms. In our analysis, the most statistically significant grouped GO terms of up-regulated or down-regulated genes in *AtSUC2-CcFT3* transgenic lines were represented in the three main GO categories: ‘biological process’, ‘molecular function’ and ‘cell component’. GO terms with corrected P-values < 0.05 were considered significantly enriched.

The highest 67 GO categories were identified as 10 different biological processes, 7 cellular components and 50 molecular functions. Amongst the 67 GO categories, biological process ‘Terpene Synthase’ was the most enriched. Of the 10 cellular components ‘Tubulin’ and ‘Microtubule Based Process’ were the most enriched. In the following 50 molecular functions, the GO categories most enriched were ‘External Encapsulating Structure’, ‘Carbon–Oxygen Lyase’, ‘Cytoskeletal Protein binding’, ‘Cell wall’ and ‘Enzyme inhibitor’ (Fig. 10B).

To position genes that had the most significant change in expression pattern in response to the transgenic expression of *CcFT3* in the flowering induction pathway, a custom MapMan mapping file was created using an *Arabidopsis* outlined pathway adapted from Kim^37^ and genes from Phytozome annotation database to compare the DEGs of non-transgenic and transgenic plants. Interestingly, the changes in expression pattern were mostly observed in pathway genes downstream of *FT* including *FT*, *FD*, *SOC1*, *FUL* (or *AGL8*) and *AP1*. There was a difference of expression in a few genes upstream of the florigen signal including: *CO*, *SVP* and *ELF4*. *CO* was upregulated in the transgenic lines compared to the non-transgenic line; however, this change was near a one-fold difference. *SVP* was downregulated in *AtSUC2-CcFT3*, which is not surprising as *SVP* is known to play a role in the downregulation of *FT*^38^. The repression of *SVP* may lead to the induction of the flowering pathway.

## Discussion

In this study, the expression of the *CcFT3* transgene was evaluated to induce early flowering in immature Carrizo citrange rootstock. Stable and uniform precocious flowering that reduces the long juvenile phase will greatly benefit citrus genetic improvement programs. This will decrease the time required to evaluate the fruit quality of potential candidates from a T1, somaclone, or transgenic population. The accumulation of the FT protein is a critical step necessary for the activation of a signaling cascade that promotes flowering in plants. Transgenic plants expressing *CcFT3* driven by a constitutive 35S promoter primarily flowered in vitro and did not survive beyond that stage. Genetic expression analysis revealed a several thousand-fold higher expression of *CcFT3* under the 35S promoter compared to the other promoter driven lines. While a high level of gene expression is desirable in some cases, this is not an efficient strategy to be used in regulating the flowering genes. The 35S promoter has also showed detrimental effect when used to drive *FT* expression in trifoliate orange (*Poncirus trifoliata* L. Raf.) causing an abnormality in the flower phenotype^3^. In our studies, the few 35S overexpressing lines that were successfully recovered never flowered and were discarded. Since detailed gene expression analysis was not performed on these lines, we speculate that these lines did not have the expression levels necessary to induce flowering or expressed the transgene in non-essential tissues such as the root or random gene silencing^39^. Heat induction can lead to the efficient expression of transgenes^40^, however *FT* could not be induced using this method. Similarly, the *NOS* promoter which is capable of functioning in the meristematic tissues^41^ did not induce *FT* in our studies, apart from the one line that flowered once.

Targeted expression of the *Arabidopsis SUC2* promoter in the phloem as described in this study or using a virus-based system for the delivery of the gene efficiently induced early flowering with no alteration of the plant architecture, leaf, flower or fruit morphology^4^. The *Arabidopsis SUC2* promoter has proven to be an excellent phloem specific promoter that has also resulted in the development of phloem specific disease resistant citrus trees^28,42^. The export of the FT protein from phloem companion cells can trigger flowering, and the *Arabidopsis SUC2* promoter can efficiently target transgene expression into those cells^30,43^. Thus, targeted expression of the FT protein in these cells was sufficient for triggering the early flowering response in our transgenic citrus lines.

The accumulation of the *CcFT3* in our transgenic lines resulted in the expression of genes involved in the flowering induction pathway similar to that reported following the endogenous *FT* gene induction^4,44^. Among the several genes upregulated in the flowering pathway following accumulation of *CcFT3* transgene, three *FT* endogenous genes were detected. In our transgenic lines, the *CcFT3* transgene was unable to induce accumulation of the *CONSTANS* (*CO*). In *Arabidopsis*, CO protein activation occurs upstream of the synthesis of the FT protein^45^ and *SOC1* is a convergence point for several genes in the flowering pathways^46^. Therefore, the absence of accumulation of CO and the induction of *SOC1* observed in our *AtSUC2-CcFT3* transgenic lines might lead to the speculation that this genes function similarly to *Arabidopsis* in citrus, however, more experiments are need to be performed before coming to this conclusion. The repression of the *TFL1* gene was observed in the transgenic lines, suggesting a possible opposing relationship between *TFL1* and *FT* gene expression compared to the relationship between *SOC1* and *FT*. Our results suggest that the *CcFT3* transgene may be capable of functioning like the endogenous *FT* gene in citrus.

Sequence analysis of orange1.1g036943m revealed this gene to be a *HEADING DATE 3A* (*Hd3a*) gene, an ortholog of *FT*. In addition, a strong homology between our transgene, orange1.1g036943m and orange1.1g041559m with 90.9% and 87.2% similarity, respectively suggesting that either of these genes can be candidates to CcFT3 homolog. Orange1.1g036943m likely functions similar to *AtSUC2-CcFT3* transgene and its induction likely results in an early-flowering phenotype^47^. Several MADS-box genes, which also participate in the flowering process^48^, were highly upregulated in our transgenic MP3 line. In *Arabidopsis*, *AGL14* is preferentially expressed in roots^49^. However, in this study, leaf tissues extractions revealed that the *C. sinensis AGL14* homolog orange1.1g042438m was expressed in *CcFT3* transgenic line leaves, similar to the findings of Perez-Ruiz et al.^50^ who reported that the overexpression of *AGL14* promoted early flowering in *Arabidopsis* transgenic lines (35S-AGL14). This demonstrates the ability of the *AGL14* to function beyond the root system^50^. *AGL6* is another MADS box gene highly induced in our *AtSUC2-CcFT3* line. Since each MADS-box protein has a specific protein–protein interaction pattern, similarities between *AGL6* and *AP1* suggest that these genes may function as activators for *FT* and *SOC1*^51^.

Enhanced expression of the *AP1* and *LFY* meristem-identity genes is the final step in the flowering pathway, resulting in floral induction^37^. *AP1* was highly induced in our transgenic plants, while *LFY* was weakly expressed. Thus, prolonged *AP1* upregulation was sufficient to bypass *LFY* requirements and alter the competence of the plant to flower. *AP1* accumulation in the meristem is necessary for flowering induction^52^ confirmed by earlier results, which indicated that *FT* can act as a redundancy gene of *LFY* to activate *AP1*^53^. A recent transcriptomic study failed to detect an upregulation of *LFY* in an early flowering coconut genotype^54^. However, it is possible that the signaling cascade may differ from the classical *Arabidopsis* flowering induction. An increase in *AP1* expression has been shown to induce early flowering phenotypes in several plant species. Precocious flowering was observed in plants with abnormal overexpression of *AP1* including: *Arabidopsis*, citrus, pear, and roses, among others^38,55–60^.

*APETALA3* along with its interacting partner *PISTILLATA* (*PI*) form a AP3/PI heterodimer complex which determines petal and stamen morphology and function in *Arabidopsis*^61^. While *AP1* both directly and indirectly controls the early expression of *AP3* and *PI*, the *AP3/PI* heterodimer directly acts in concert with other factors to restrict the expression of *AP1* during early stages of floral development, thereby acting as a transcriptional/translational feedback loop (TTFL) in the apical meristem or in floral tissues^62–64^.

Genes from *AP2*/*ERF* family were also upregulated in our transgenic line. Since timing of flowering is generally induced upon environmental and stress cues, such as cold, drought, salinity, and pathogen infection^65–67^, it is likely that the induction of *AP2*/*ERF* is involved in the activation of downstream signals to maximize reproductive success.

Although JA delays flowering through repression of *FT* expression^68^, some of the important components of JA signaling pathway (JAZ1 and JAZ10) were also upregulated in our RNAseq data. JAZ1 is known to interact with *TARGET OF EAT 1&2* (*TOE1, TOE2*). These proteins induce late flowering phenotypes upon the transcriptional repression of *FT*. When the JAZ1 protein forms a complex with TOE1 and TOE2, these proteins become unavailable to repress *FT* relieving the repression effect of *TOE1* on *FT* transcription^68^. This indicates that JAZ1 may act as an allosteric inhibitor of TOE1. Similarly, JAZ10 plays an important role in the flowering regulatory process, but there may be potential redundancies between this gene and others in the JA-signaling cascade^69^.

Since RNAseq data reveals a broad picture of expression results, RT-qPCR indicating a specific fold change upon the expression of the transgene was performed to increase data reliability. In this experiment, the same RNA samples that was sequenced were also used to make cDNA for use in RT-qPCR. The upregulated citrus genes evaluated in this analysis were mapped to their *Arabidopsis* ortholog AGL*14*, *AGL8*, *AP1*, *ERF38*, *AGL8* or *WRKY72* using the phytozome and *Arabidopsis* databases. At least four of these genes, *AGL14*, *AGL8*, *AP1* and *AGL6* are directly associated with the flowering induction pathway^70^ and their overexpression in *Arabidopsis* results in an early flowering phenotype^50^. In addition, the overexpression of *WRKY72* in rice has also led to induction of precocious flowering^71^. *ERF38* has a more indirect effect in flowering phenotype since it is associated with abiotic stress that eventually result in early flowering^72^. The downregulated genes assayed in this study were mapped as *AP2, AP3, AGL11, GA2OX8, HSP70*, and *WRKY23*. *WRKY23*, *AP2* and *AP3* have been associated with late flowering phenotypes^73^. The *HSP* family of proteins are widely known to play role in abiotic stresses and some of the *HSP* have a potential to induce the late flowering phenotype in higher plants^74^. The gibberellin (GA) pathway is one of the main pathways in *Arabidopsis* involved in flowering induction. In *Arabidopsis*, GAs could promote flowering by activating the expression of *LFY*^75^. Accordingly, the downregulation of *GA2OX8* in our *CcFT3* transgenic lines could act like the *AtGA2OX8* of *Arabidopsis* that 2β-hydroxylates the C_20_-GA precursors resulting in a decrease of active GAs levels^76^, thereby inducing a GA-deficient phenotype such as delayed flowering in different species.

*FT* induction was stable in our transgenic lines and could be transmitted to the seedling progeny, which also flowered within a year of germination. Trees grew normally, produced normal flowers with viable pollen, and these trees had stable *FT* expression throughout the five years of this study. Additionally, expression of the *CcFT3* transgene resulted in early flowering *Arabidopsis* plants and the FT protein could be induced through the graft union resulting in precocious flowering on one-year old budded ‘Valencia’ sweet orange shoots. Thus, these *FT* lines have great potential in shortening the breeding cycle. Stable transmission of the early flowering phenotype from the rootstock to the scion can alleviate many of the long juvenility issues that hampers the rapid development and evaluation of citrus cultivars.

## Conclusion

Our results revealed that the phloem-localized expression of the *CcFT3* gene sustained precocious flowering in Carrizo citrange rootstocks. Flowers were produced in transgenic lines as early as 16 months from transformation in the rootstocks. Transcriptomic data indicated that the expression of the *CcFT3* gene triggered several critical genes in the flowering process. Altogether, our results provide definitive evidence that the *CcFT3* transgene when expressed in phloem cells can induce early flowering in citrus.

## Material and methods

### Plasmid vector construction and transgenic plant production

The *Citrus unshiu FT1*, *FT2* and *FT3* sequences available in the NCBI database (AB027456, AB301934 and AB301935) were utilized to find the homologous sequences from the *C. clementina* genome in the Phytozome database (https://phytozome.jgi.doe.gov). The *CcFT1* and *CcFT3* DNA sequences (NCBI accession nos: MT707614 and MT602515) were amplified by PCR from *C. clementina* ‘Nules’ cDNA and modified to remove internal restriction sites to facilitate cloning using the Vector NTI (Life Technologies, NY, USA) bioinformatics software. A *BamHI* site was inserted immediately upstream of the translation start site and a *SacI* site was added following the stop codon. The sequences from all the clones were verified using Sanger sequencing and cloned into the complementary sites of the pCAM-CLON plant transformation vector. An *Arabidopsis thaliana HSP* (*AtHSP18.2*) promoter^29^, an *AtSUC2* promoter^28^, or a *NOS* promoter^77^ were used to replace the 35S promoter in the pCAM-CLON vector. In total, eight *FT* based constructs were evaluated in this study (Supplementary Figure S2). The gene sequence of *CcFT*3 was fused in frame to the *egfp* gene (NCBI accession no: MT602516) to create a *CcFT3-EGFP* fusion gene. The fusion gene was artificially synthesized (Twist Bioscience, San Francisco, CA) and similarly cloned into the pCAM-CLON plant transformation vector.

Epicotyl explants of in vitro grown nucellar seedlings of Carrizo citrange (*Citrus sinensis* Osb. X *Poncirus trifoliata* L. Raf.) were used in all *Agrobacterium* genetic transformation experiments as outlined by Dutt and Grosser^78^. Putative transgenic lines that rooted in a kanamycin supplemented citrus rooting medium (RMM) was transferred to a peat-based commercial potting medium (Metromix 930, Sun Gro Horticulture, WA, USA) and acclimated under a 75% shade cloth in a greenhouse at 32° ± 4 °C. Trees transformed with the *AtHSP18.2* promoter were incubated in a growth chamber at 37 °C for 3 h following transfer to the greenhouse. This cycle was repeated at least 6 times in weekly intervals.

### Molecular analysis of transformants

PCR was carried out on all putative *CcFT3* lines with *AtSUC2-FT3*-F and *AtSUC2-FT3*-R primer set to amplify a 700 bp fragment, partly from the 3′ end of the *AtSUC2* promoter and the 5′ of the *CcFT* transgene and *NPTII*-F and *NPTII*-R primers to amplify a 500 bp *nptII* fragment (Supplementary Table S1). Putative transgenic plants were verified by PCR using gene specific primers and an Extract-N-Amp Plant PCR Kit (Sigma-Aldrich, St. Louis, MO). The presence of transgenes was confirmed by visualizing amplicon bands at the expected size on a 1% agarose gel containing GelRed (Biotium, Hayward, CA) under ultraviolet light. All images were recorded and digitized using an Axygen Gel Documentation System (Corning Incorporated, Tewksbury, MA, USA).

A Southern blot was performed to confirm the stable integration of the gene fragment and to determine the copy number in selected *CcFT3* transgenic plants. Genomic DNA was extracted from young leaves using a cetyl trimethylammonium bromide (CTAB) protocol^79^, and was enzymatically digested using *EcoRI*, to cut within the T-DNA, but outside of the probe region. After overnight electrophoresis in 0.8% agarose gel at 25 V, DNA fragments were denatured, depurinated, and blotted onto Hybond N + membrane (Roche, Indianapolis, IN) according to manufacturer’s protocol. The digoxigenin (DIG) labelled *FT3* probe (Roche, Indianapolis, IN) was UV cross-linked, and hybridized to the membrane and processed following the manufacturer’s instructions. The *FT3* probe was synthesized by PCR using a DIG labelling kit (Roche). The amplification of amplicons by PCR was performed using the primers listed in the Supplementary Table S1 under the following cycling conditions: 2 min at 95 °C, 30 cycles of 30 s at 95 °C, 30 s at 66 °C, 40 s at 72 °C and a final extension of 10 min at 72 °C, yielding a 525 bp product. Finally, the blot was exposed to a Kodak X-ray film for 30 min prior to image analysis.

### RNA extraction, cDNA synthesis and quantitative PCR

Citrus RNA was isolated either from 100 mg of leaf blades or leaf petioles using a Direct-zol RNA Miniprep Plus Kit (Zymo Research, Irvine, CA) according to the manufacturer’s protocol. The cDNA was synthesized using RevertAid First Strand cDNA Synthesis Kit (Thermo Fisher Scientific, Waltham, MA) according to the manufacturer’s instructions. Transcripts were quantified by real-time quantitative PCR (RT-qPCR). The RT-qPCR reaction mix consisted of SYBR Green PCR Master Mix (Applied Biosystems, Foster City, CA) and primers as outlined in Supplementary Table S1 for a 20 μL reaction. The reaction conditions consisted of initial denaturation at 95 °C for 30 s, followed by 40 cycles of 95 °C for 15 s and 60 °C for 1 min. A no template/water control and non-transgenic cDNA were used as RT-qPCR negative controls. The relative mRNA levels were compared to those of the endogenous *C. sinensis ACTIN* gene^80^ and calculated using the 2^-ΔΔCT^ method^81^.

### Protein extraction and immunoblot analysis

Protein analysis was carried out by western blotting. Approximately 100 mg of young citrus leaves were flash frozen in liquid nitrogen and crushed using chilled mortars and pestles. The tissue was homogenized with 500 μL of CelLytic P Cell Lysis Reagent containing protease inhibitors (Sigma-Aldrich Corp., St. Louis, MO). The protein extract was transferred to a microcentrifuge tube and centrifuged 17,949×*g* for 10 min at 4 °C. The supernatant was transferred to another microcentrifuge tube, and this process was repeated three times. The protein concentration was measured using a Quick Start Bradford protein assay (Bio-rad Laboratories, Inc, Hercules, CA). Crude protein was separated on a 12% SDS/PAGE gel cast from TGX FastCast Acrylamide Kit (Bio-Rad) and transferred to a PVDF membrane. The PVDF membrane was incubated for 5 min in Ponceau-S solution (40% methanol (v/v), 15% acetic acid (v/v), 0.25% Ponceau-S), and subsequently de-stained with deionized water before the membrane was photographed. The Ponceau-S solution was incubated in tap water until complete removal of the dye, before blocking the membranes in 5% dry milk prepared in 1 × TBST for 30 min. The membrane was probed with a CcFT3 polyclonal antibody (ABclonal, Woburn, MA) and incubated overnight with agitation at 4 °C. The membranes were washed three times using 1 × TBST and then probed with a secondary antibody (goat anti-rabbit IgG Antibody, house radish peroxidase (HRP)-conjugate). Immunoblots were developed using an ECL detection kit and imaged using X-ray film autography (Thermo-Fisher Scientific, Waltham, MA).

### Citrus propagation and precocious flowering phenotype assessment

The *AtSUC2-CcFT3* number 3 transgenic line (MP3) was selected for further analyses. Single node cuttings were produced from two- and half-year-old trees at the time of propagation, in a mist bed. Non-transgenic Carrizo citrange cuttings of a similar age were used as controls. Budwood from a 1-year old ‘Valencia’ sweet orange seedling were grafted onto six-month-old clonally propagated rootstocks. Precocious flowering phenotype evaluation was performed weekly. Leaf samples for RNA isolation were collected periodically. The budded plants were photographed for documentation.

### Microscopy

Pollen was collected from anthers of the MP3 transgenic line and non-transgenic controls and placed on a slide. Pollen grains were incubated in 50% Gram’s iodine solution diluted with deionized water on a slide for 5 min, or a 1% solution of 2,3,5-Triphenyltetrazolium chloride (TTC) on a microscope slide for two hours, before imaging. Controls were also examined as described above by incubating pollen grains in deionized water.

For SEM images of pollen grains, anthers were collected from fully expanded, unopened flowers and fixed in a 4% paraformaldehyde solution in 1X PBS. Anthers were rinsed three times for 10 min in 1X PBS and dehydrated in an ethanol series (30%, 50%, 70%, 80%, 90%, 95%, 100%, 100%, 100%). Samples were critical point dried using a Ladd 28000 critical point dryer (Ladd Research Industries, Williston VT, USA). Anthers were cut open and pollen was retrieved and placed on a double-sided 12 mm Carbon sticker (Electron Microscopy Sciences, Hatfield PA, USA). The samples were sputter coated with palladium/gold using a Ladd 30800 sputter Coater (Ladd Research Industries). Pollen grains were observed, and images were recorded using a Hitachi S4000 scanning electron microscope (Hitachi, Tokyo, Japan).

### Transcriptome profiling of *AtSUC2-CcFT3* transgenics

RNA extracted from three technical replicates of MP3 transgenic lines and control non-transgenic plants were sequenced using the Illumina NovaSeq 6000 platform attuned for a 2 × 150 read length configuration. To obtain clean reads, adaptors were removed by AdapterRemoval (version v2.2.2, https://github.com/MikkelSchubert/adapterremoval)^82^. The reads were filtered based on quality and length using Trimmomatic (version v0.39, http://www.usadellab.org/cms/?page=trimmomatic)^83^ by removing the adaptors and reads with less than 100 bases and an average quality of 16 or less. The following parameter were applied: A minimum length of 100 bases, a trailing and leading of 16, and a sliding window of 16:25. A final inspection of the reads was carried out by using FastQC (version v0.11.8, https://www.bioinformatics.babraham.ac.uk/projects/fastqc/) in order to ensure proper cleaning was performed^84^. The raw data has been deposited into the National Center for Biotechnology Information’s Sequence Read Archive (https://www.ncbi.nlm.nih.gov/sra) with a SRA accession number: PRJNA668159.

### Mapping of the reads, transcript count and differential expression analysis

The cleaned reads were mapped to the *C. sinensis* genome using STAR^85^. The *C. sinensis* genome and annotation used in the mapping were obtained from Phytozome^86^. The STAR output bam files were sorted and indexed by SAMtools (version v1.7, https://github.com/samtools)^87^. Read counts of each gene from the sorted and indexed bam files were counted by FeatureCounts v1.6.0^88^. The outputted read counts for each gene and sample were placed into a comma-separated values format master file. The construction of a metadata file was also performed, which contained the samples names and trial type.

To obtain the differentially expressed genes (DEGs), DESeq2 v3.10 was used^89^. The metadata file and counts master file were inputted into DESeq2. The counts for the comparison were normalized and then estimated dispersion followed. The output list of DEGs were then filtered to remove any DEGs without a |log^2FoldChange^|≥ 1 and a P adjusted value (FDR) ≤ 0.05.

### Go enrichment and pathway analysis

The list of statistically significant and high log^2FoldChange^ DEGs were analyzed using agriGO (version v2, http://systemsbiology.cau.edu.cn/agriGOv2/)^90^ in order to obtain a significant grouped Gene Ontology (GO) terms. A significance level of p-value (P < 0.05) was selected as the cutoff in agriGO v2. The statistically significant GO terms were then run with REVIGO (http://revigo.irb.hr/) to remove redundant GO terms^91^. MapMan (version v3.5.1, https://mapman.gabipd.org/mapman) was then used to analyze the DEGs and for a more in-depth analysis on individual pathways and other factors^92^.

The filtered DEGs from DESeq2 had their PacID attached and were then imported into MapMan. A custom MapMan mapping file and pathway were generated using genes from the photoperiod, vernalization, autonomous and gibberellin pathways acquired from the *Arabidopsis* and Phytozome annotation databases. The DEGs with the PacID attached were compared to the custom MapMan file generated to show the changes between non-transgenic and transgenic plants.

### Confocal microscopy

Protein expression and localization assays were performed using *CcFT3-EGFP* fusion driven by 35S and *AtSUC2* promoters. The clones were transiently expressed in *Nicotiana benthamiana* plants according the method described by Sparkes et al.^93^ with minor modifications. A single colony of *Agrobacterium* strain EHA105 containing each construct was grown in Luria–Bertani broth containing rifampicin (25 mg/L) and kanamycin (100 mg/L) incubated at 28 °C overnight. The culture was centrifuged and re-suspended in infiltration buffer (10 mM 2-(N-morpholino) ethanesulfonic acid (MES), pH 5.85; 10 mM MgCl_2_) containing 200 µm acetosyringone. The bacterial suspension was incubated at room temperature for 3–4 h and the leaves were infiltrated with bacteria using a 1 mL needleless syringe. Leaves were imaged 3 days post-infiltration. Leaf discs were punched using a hole puncher, mounted on a slide and imaged using a Leica SP8 laser-scanning confocal microscope (Leica Microsystems Inc., Buffalo Grove, IL, USA).

## Supplementary Information

Supplementary Information
