# Supplementary material for: The vascular targeted citrus FLOWERING LOCUS T3 gene promotes non-inductive early flowering in transgenic Carrizo rootstocks and grafted juvenile scions
Source: Sci Rep. 2020 Dec 8;10:21404. doi: 10.1038/s41598-020-78417-9 (PMC7722890; doi:10.1038/s41598-020-78417-9)
Supplement: Supplementary file 1 — Supplementary Information [file 41598_2020_78417_MOESM1_ESM.pdf]

Supplementary Information

The vascular targeted citrus *FLOWERING LOCUS T3* gene promotes non-inductive early flowering in transgenic Carrizo rootstocks and grafted juvenile scions

Juliana M. Soares[1], Kyle C. Weber[1], Wenming Qiu[1,2], Daniel Stanton[1], Lamiaa M. Mahmoud[1,3], Hao Wu[1], Patrick Huyck[1], Janice Zale[1], Kawther Al Jasim[1,4], Jude W. Grosser[1] and Manjul Dutt[1]*

[1] Citrus Research and Education Center, University of Florida, Lake Alfred, FL, USA 33850; [2] Institute of Fruit and Tea, Hubei Academy of Agricultural Sciences, Wuhan, China 430064; [3] Pomology Department, Faculty of Agriculture, Mansoura University, Egypt; [4] Horticultural Sciences Department, College of Agriculture, Al Qassim Green University, Babylon, Iraq

* Corresponding author: manjul@ufl.edu

Supplementary Table S1: Primers used in this study.

Seq Name / Accession number		Primer sequences 5'→3'
NPTII_PCR	Forward	CGGTGCCCTGAATGAACT
	Reverse	GCCAACGCTATGTCCTGATA
AtSUC2-CcFT3_PCR	Forward	ACACGTGTCACGAAGATACC
	Reverse	CGGAGGTCCCAGATTGTAAAG
orange1.1g042438m	Forward	TGGAGCTGAAGAGGATAGAGAA
	Reverse	CCAGAGGGAGAGAAGATGACTA
orange1.1g035470m	Forward	GAAGGGTGCAGCTGAAGAG
	Reverse	GGAGAAGACGATCAAAGCAACT
orange1.1g042642m	Forward	CTTGCTGGTAAAGCAGATTAAGG
	Reverse	GGTGGCTGTGGAAGTAGAA
orange1.1g023148m	Forward	ACTGCTAGCGATAACGATCAC
	Reverse	GGCGCATTCTTACTCCTCTATAA
orange1.1g037149m	Forward	AGTGGAGCTGAAGAGGATAGA
	Reverse	CCACGGCTAGAGAAGATGATAAG
orange1.1g009794m	Forward	AGGATTACCAGACCCTTCAAAC
	Reverse	TCTTCCGAGGCTAAGAGATACA
orange1.1g041209m	Forward	GCCTACTGCTGCTGCTATT
	Reverse	AAATCCTCGCCTCCAAGATG
orange1.1g019949m	Forward	TTACCCAACCCTCTTCCATAAC
	Reverse	GCGAGCAAGATCAATCAAAGG
orange1.1g036452m	Forward	GCAGCAGGAAGAGAGGAATTAT
	Reverse	AGAAGGTCACTTGGCGATTT
orange1.1g032690m	Forward	TCATCCATGAAACTAGGGATCAA
	Reverse	CATCTTCTGGCAACACCAATC
orange1.1g026276m	Forward	CGAATGGAAGCCCTCAATCT
	Reverse	TCTTCACTGTACGAGGTTTCAC
orange1.1g040046m	Forward	GAGGAAAGATCCAGATCAAGAGG
	Reverse	CATCACAGAGAACGGTGAGTT
CcFT3	Forward	CCCAGCCTTAGGGAGTATTTG
	Reverse	GGCCTTGGGCTTTCATAGT
CsCO	Forward	TGACCAGGACGAGGAAGAA
	Reverse	CCGTGCTGTTGCTGATTATTG
CsSOC1	Forward	TGCTGAGGTTGCCGTTATT
	Reverse	CCAGACCTTCTCCCAATAGTTTC
CsTFL	Forward	CTTTCCGTCCACAGTTGTTTC
	Reverse	ATCTCGTAGCTCACCAATTCC
CsAP1	Forward	CAGAAAGACCAAAGGCACTATTT
	Reverse	CAGCCTTCTCTCTCTCCTTAATC

CsLFY	Forward	AGCTTTCACGGCGAGTTT
	Reverse	CGCTGCTGTGTGGTATCTTAT
CsActin	Forward	GCTGCCTGATGGCCAGATC
	Reverse	AGTTGTAGGTAGTCTCATGAA
FT3_probe	Forward	GTGTCACGAAGATACCCTACGC
	Reverse	CCCCTGTGGTTGCTGGAATATC

Supplementary Table S2: Summary of sequencing, cleaning, and mapping of reads

Run Name	Raw read count	Read count after cleaning	Surviving Read Percent	Mapped reads	Mapped reads percent
WT-1	22,299,060	21,226,809	95.19%	17,528,606	82.58%
WT-2	21,585,699	20,540,415	95.16%	16,813,253	81.85%
WT-3	22,326,759	21,175,690	94.84%	17,475,408	82.53%
WT Average	22,070,506	20,980,971	95.06%	17,272,422	82.32%
FT3-1	22,600,650	21,522,068	95.23%	17,497,999	81.30%
FT3-2	22,065,043	21,098,887	95.62%	17,256,897	81.79%
FT3-3	22,813,145	21,774,061	95.45%	17,679,770	81.20%
FT3 Average	22,492,946	21,465,005	95.43%	17,478,222	81.43%

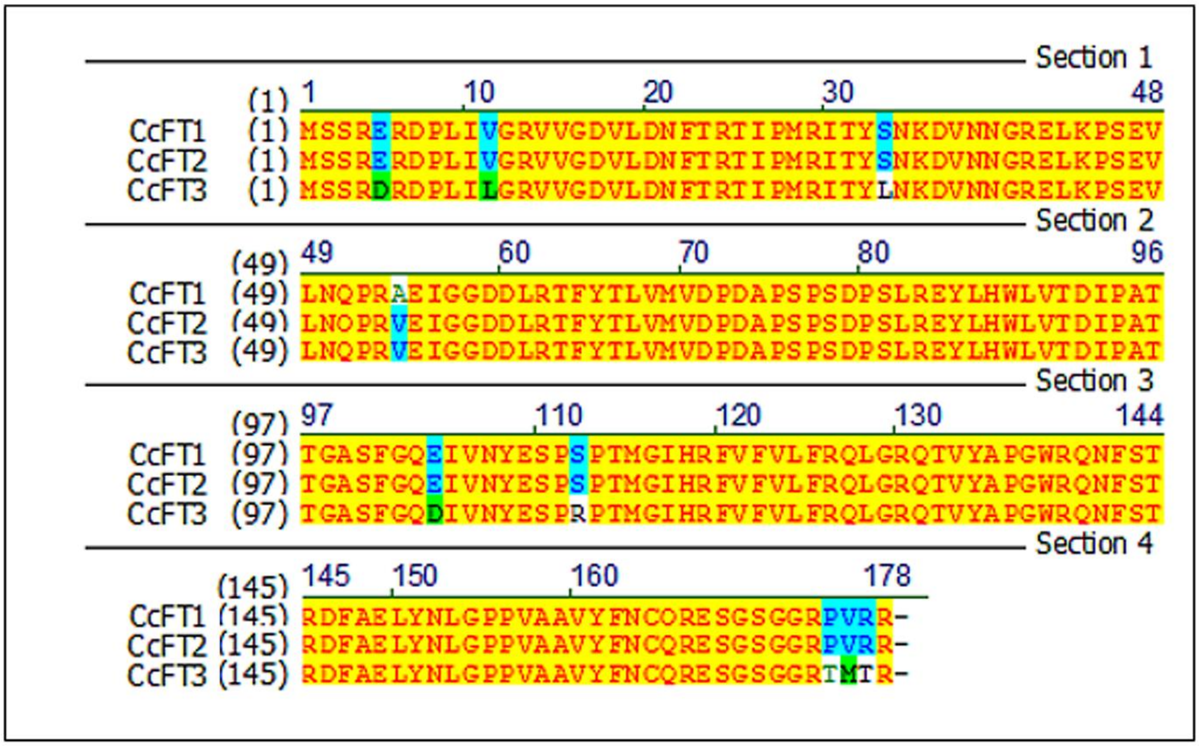

Supplementary Fig. S1. Alignment of amino acid sequences among CcFT1, CcFT2, and CcFT3 homologs.

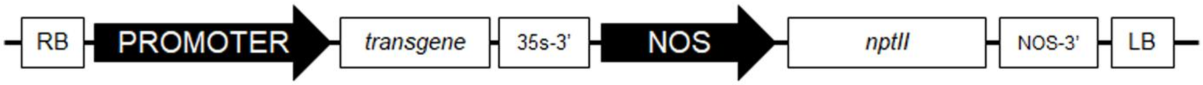

Transgene evaluated:

- CcFT1 – Citrus clementina FT1
- CcFT3 – Citrus clementina FT3

Promoter evaluated:

- 35S - Cauliflower mosaic virus (CaMV) 35S
- AtHSP – Arabidopsis thaliana Heat Shock Protein (HSP)-18.2
- AtSUC2– Arabidopsis thaliana SUC2 sucrose-H+ symporter
- NOS - Agrobacterium tumefaciens nopaline synthase (nos)

Supplementary Fig. S2. Schematic representation of T-DNA region of the eight binary vectors used in this study.

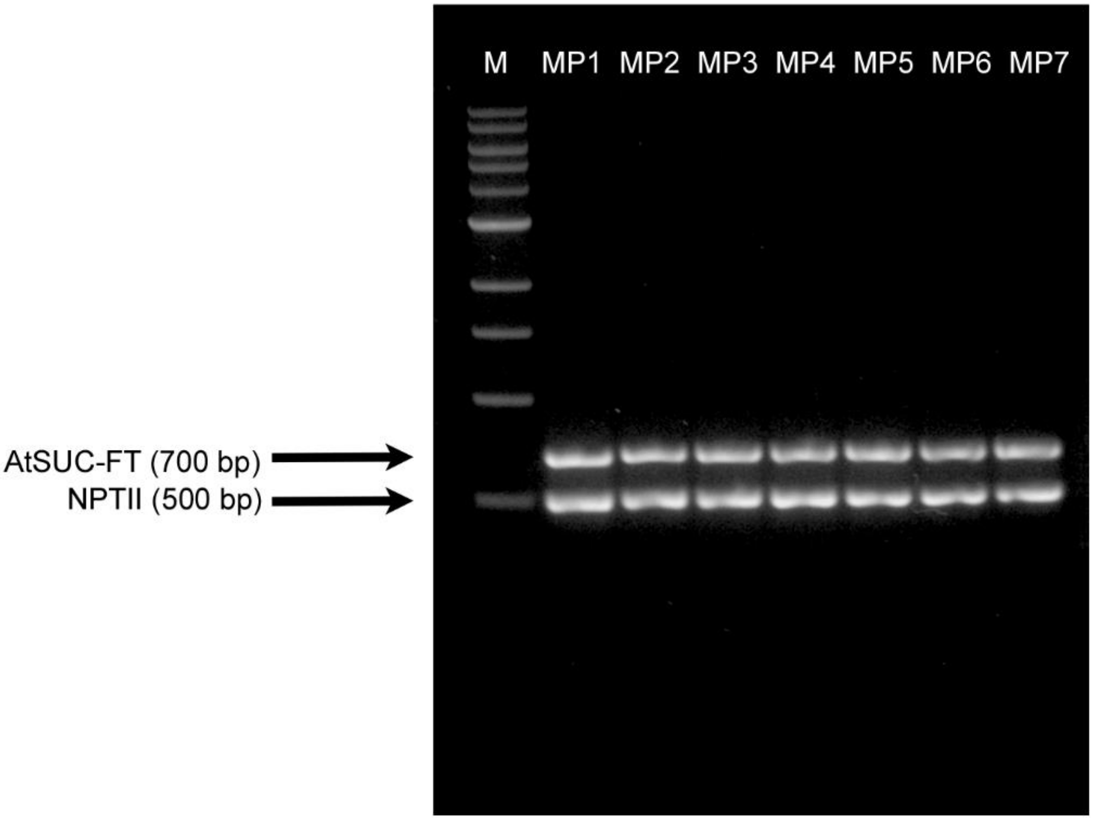

Supplementary Fig. S3. Amplification products obtained from duplex PCR of *AtSUC2-CcFT3* transgenic Carrizo genomic DNA with gene-specific oligonucleotide primers. A 700 bp fragment, consisting of the 3' of the AtSUC2 promoter and 5' of the *FT* gene was amplified along with a 500 bp fragment of the *nptII* gene. M, 1 kb marker; 1–7 are seven individual early flowering transgenic lines.

Arabidopsis transformation

Arabidopsis non-transgenic seeds were sown on a PRO-MIX soilless medium (Premier Tech Horticulture, Quakertown, PA) and the seeds were vernalized for 2-3 days at 4°C to achieve synchronized germination. The plants were grown in growth chambers (Percival Scientific, Spring Valley, IA) maintained at 22°C, 65% relative humidity with a 16 h photoperiod. The non-transgenic plants were subsequently transformed with *Agrobacterium tumefaciens* strain EH105 containing a pCAM-CLON plant transformation vector harboring the *CcFT3* gene using the floral dip technique[81]. *Agrobacterium* cells were streaked on Luria-Bertani (LB) medium plates supplemented with kanamycin (100 μg/mL) and rifampicin (25 μg /mL) and incubated at room temperature for two days. From this plate, a single colony was cultured overnight in 5 mL of LB liquid medium at 28°C with constant shaking. Approximately 3 mL of the overnight culture was inoculated into 300 mL of LB under continual shaking overnight at 28°C. The culture was centrifuged for 10 min at 1,878 x g to pellet the cells and re-suspended in transformation buffer (half strength Murashige and Skoog [MS] basal salt mixture, 3% sucrose with 0.5 mL/L of Silwett–77, pH 5.7). The transformation buffer was poured into magenta boxes and flowers were dipped (pot upside-down) into the transformation buffer for 20-30 seconds. The floral dipped plants were kept under a plastic dome overnight to maintain humidity and subsequently placed in a 22°C growth chamber. After 4 to 6 weeks, the seeds were harvested, surface-sterilized (70% ethanol for 1 min, 10% bleach for 10-15 min in a rotary shaker and washed 4-5 times with sterilized water) and plated into half strength MS medium supplemented with kanamycin (100 μg/mL). The positive transformants were transplanted in soil and used for subsequent analyses.

Results

The *CcFT3* transgene was evaluated for its ability to accelerate flowering in a heterologous species. We transformed *Arabidopsis thaliana* (Col-0) with *Agrobacterium* harboring the *CcFT3* vectors expressing the transgene either under a 35S or the *AtSUC2* promoter. Approximately one week after transplanting the T1 seedlings from an *in vitro* selection medium to soil, one flower per transgenic plant was observed (Supplementary Fig. S4 upper panel). In contrast, the non-transgenic Col-0 plants did not produce any

flowers in that given time frame. After two weeks, plants in which the *CcFT3* was driven by the 35S promoter produced several flowers per plant and in *AtSUC2-CcFT3* lines only one flower was present. However, the stem increased in length compared to the 1-week-old plants (Supplementary Fig. S4 lower panel).

Quantitative PCR (RT-qPCR) indicated that the *CcFT3* transcript accumulated to a high degree in the transgenic lines but was absent in the non-transgenic plants (Supplementary Fig. S4). Western blotting using CcFT3 specific antibody showed a high level of CcFT3 protein expression in the transgenic lines, but absent in non-transgenic Col-0 plants (Supplementary Fig. S4).

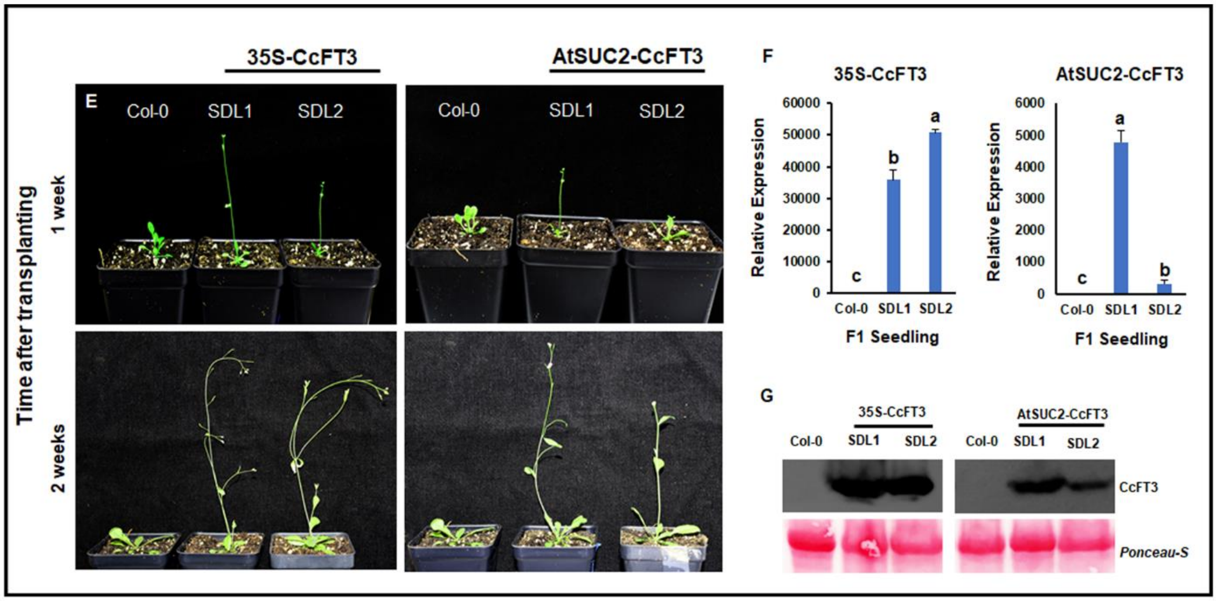

Supplementary Fig. S4. Molecular analysis of Arabidopsis transgenic lines expressing *CcFT3* under control of the 35S and *AtSUC2* promoter. (A) Flowering phenotype in *Arabidopsis* transgenic seedlings (SDL) 1 and 2 weeks after transplantation compared to the Col-0 non-transgenic control. For each transgenic and control plant, eight replicates were evaluated (B) The transcript quantification in leaf tissues of *CcFT3* transgene expressed under 35S or *AtSUC2* promoter. *Actin* was used as the reference gene. Data represent the mean (± standard deviation, SD) of three technical replicates. Different letters represent a significant difference at $P < 0.05$ using Student's t-Test. (C) Western blots of samples extracted 2 weeks after transplantation probed with CcFT3 specific antibody to visualize CcFT3 protein levels expression in the *35S-CcFT3* and *AtSUC2-CcFT3* transgenic lines. *Ponceau-S* was used as loading control. The space between transgenic and WT sample indicates the place where the blot was cut.

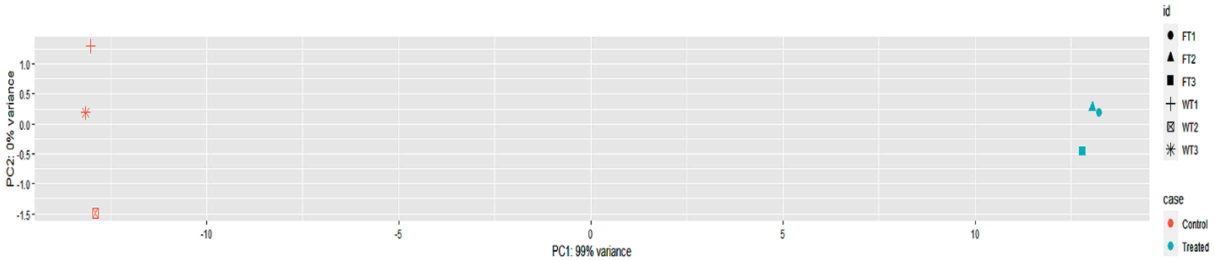

Supplementary Fig. S5. Principal component analysis (PCA) in two dimensions. RNA-Seq expression data showing the variance between the conditions, flowering transgenic and wild type non-transgenic, and the variance between the three samples per condition**.**

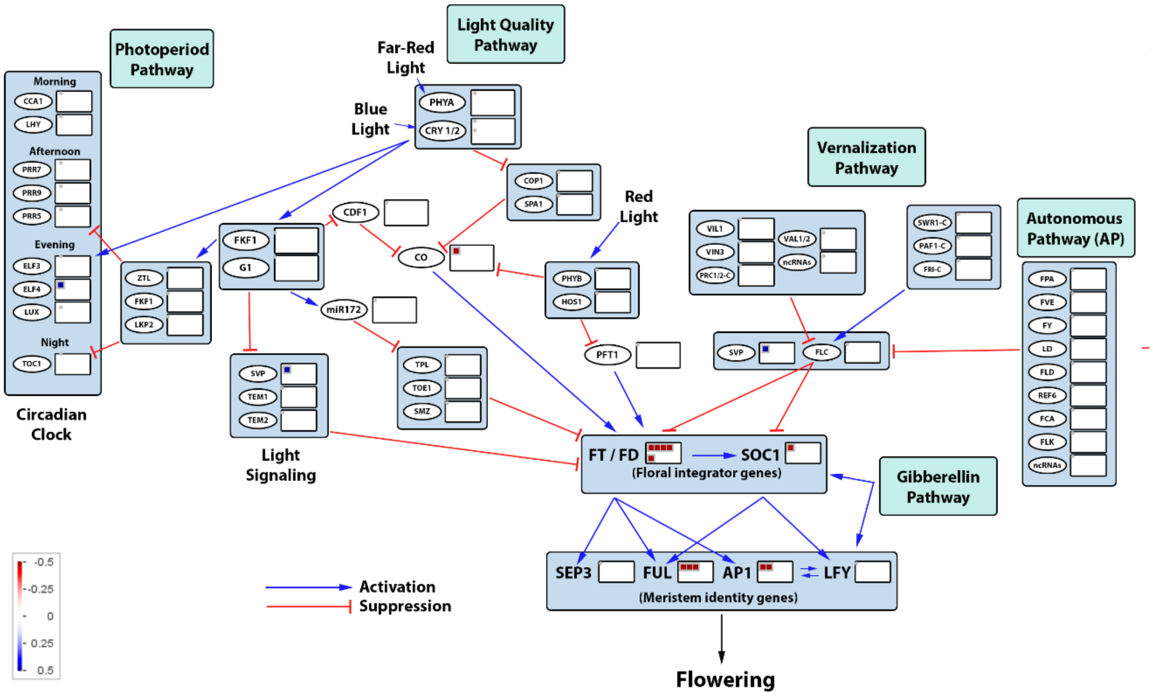

Supplementary Fig. S6. Detailed schematic representation of the multiple pathways induced in plants during flowering (adapted from Kim, 2020). The components of the flowering pathways shown are from *Arabidopsis* research. To initiate flowering transition, FT and FD proteins form a FT-FD dimer that triggers expression of SOC1 and the meristem identity genes *SEP3*, *FUL*, *AP1*, and *LFY*. *FLC* and *CO* have opposite functions suppressing and inducing *FT* and *SOC1* transcription, respectively. The photoperiod pathway is comprised of four complexes and is tightly connected to the light quality pathway. GI and light quality pathway operate as both CO-dependent and CO-independent pathways. CO protein activity can also be either suppressed by PHYB or enhanced by PHYA, CRY1, and CRY2. PFT1 mediated activation of FT can be suppressed by PHYB affecting FT transcription. The autonomous pathway is comprised of nine components which represses FLC and SVP. The gibberellin pathway directly affects the transcription of *SOC1* and *LFY*. The vernalization pathway includes multiple complexes that can activate and repress *FLC*. The red bar indicates the suppression or negative effect and the blue arrows indicate the activation or positive effect. The red and blue squares represent genes that were induced and repressed, respectively in the *AtSUC2-CcFT3* MP3 transgenic line.

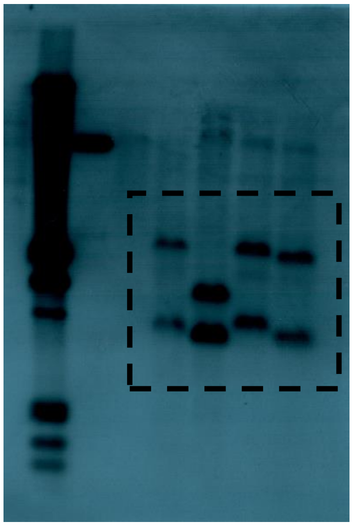

Supplementary Fig. S7. Uncropped original Southern blot image of Fig. 4A

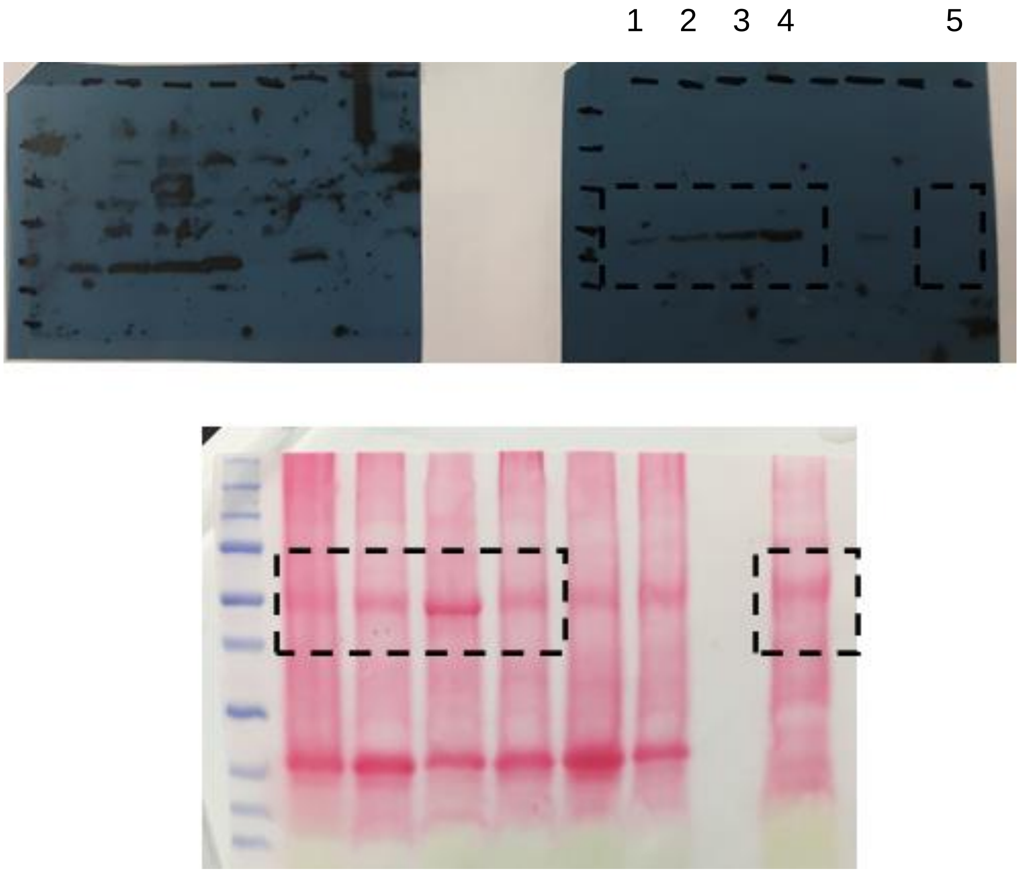

Supplementary Fig. S8. Uncropped original Western blot images of Fig. 5C. Different exposure imaged films are indicated in top panel. Ponceau-S stained membrane in the bottom. Lanes were loaded as following: (1) AtSUC2-CcFT1, (2) AtSUC2-CcFT2, (3) AtSUC2-CcFT3 (4) AtSUC2-CcFT4 and (5) non-transgenic.

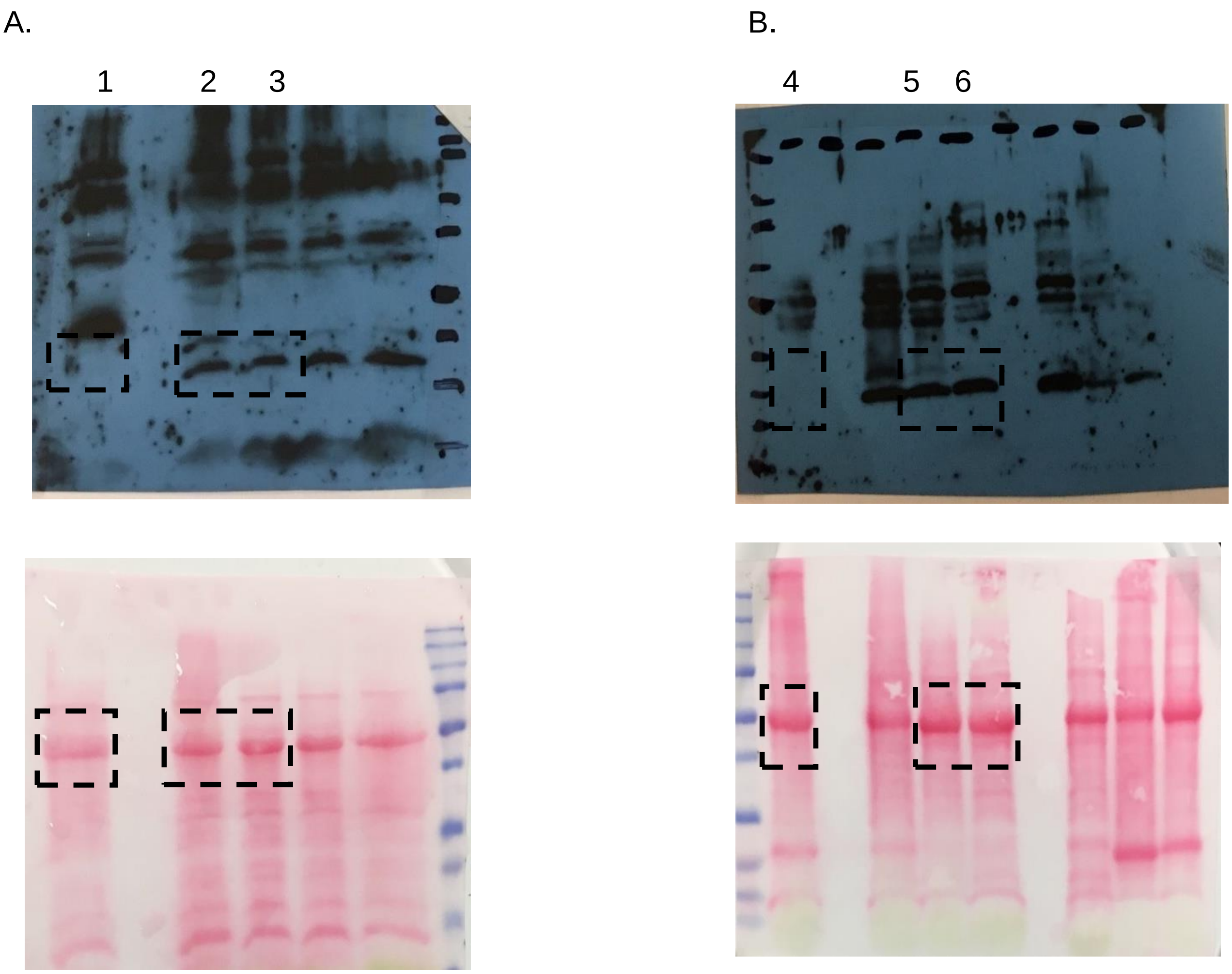

Supplementary Fig. S9. Uncropped original Western blot image of Fig. 5D showing non-transgenic and progeny of *AtSUC2-CcFT3* (A) MP1 and (B) MP2. Ponceau-S stained membrane in the bottom. Lanes were loaded as following: (1) non-transgenic (2) AtSUC2-CcFT1 seedling number 1, (3) AtSUC2-CcFT1 seedling number 2, (4) non-transgenic, (5) AtSUC2-CcFT2 seedling number 1 and (6) AtSUC2-CcFT2 seedling number 2.

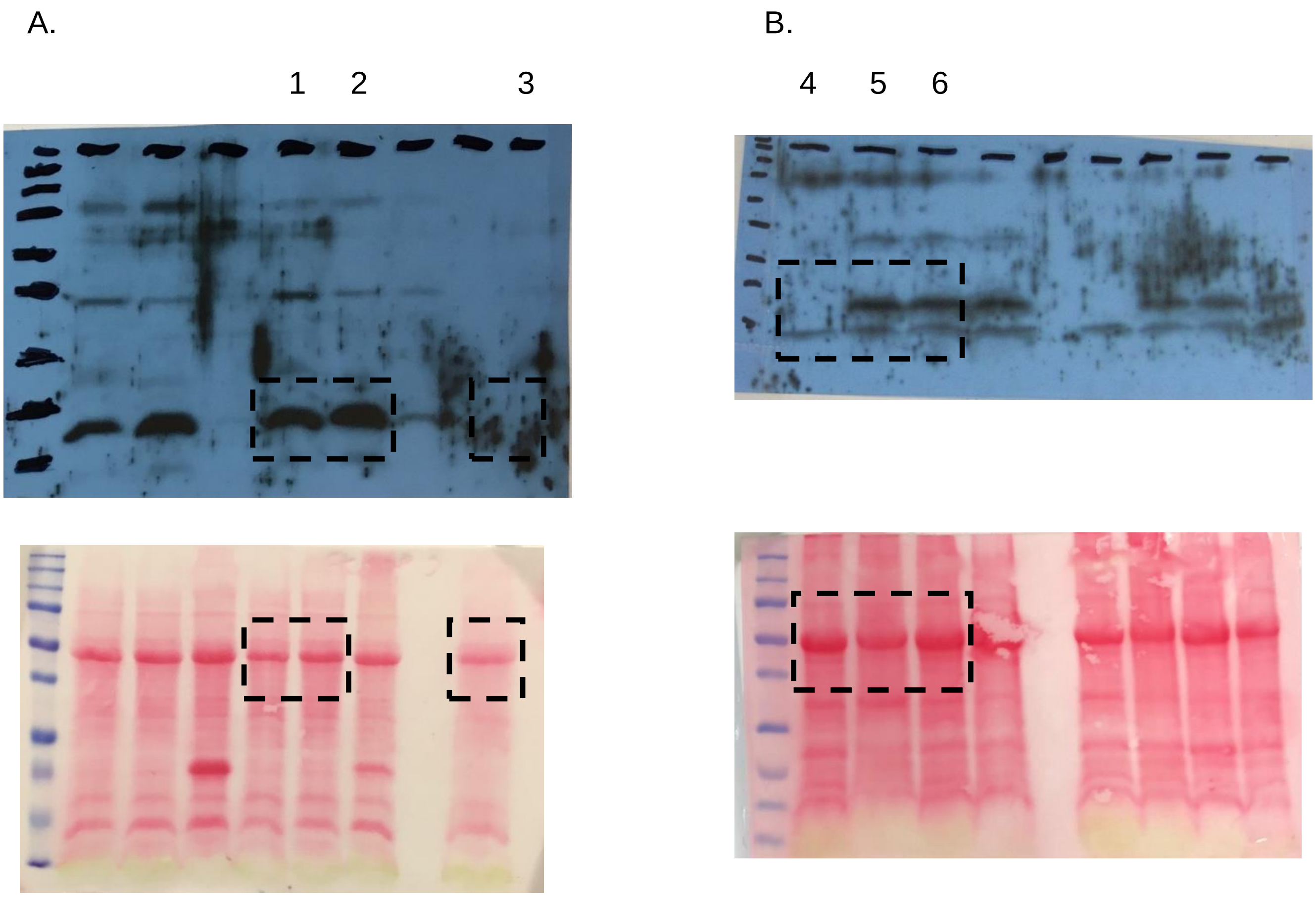

Supplementary Fig. S10. Uncropped original Western blot images of Fig. 5D non-transgenic and progeny of *AtSUC2-CcFT3* (A) MP3 (B) MP4. Ponceau-S stained membrane in the bottom. Lanes were loaded as following: (1) AtSUC2-CcFT3 seedling number 1, (2) AtSUC2-CcFT3 seedling number 2, (3) non-transgenic, (4) non-transgenic, (5) AtSUC2-CcFT4 seedling number 1 and (6) AtSUC2-CcFT4 seedling number 2.

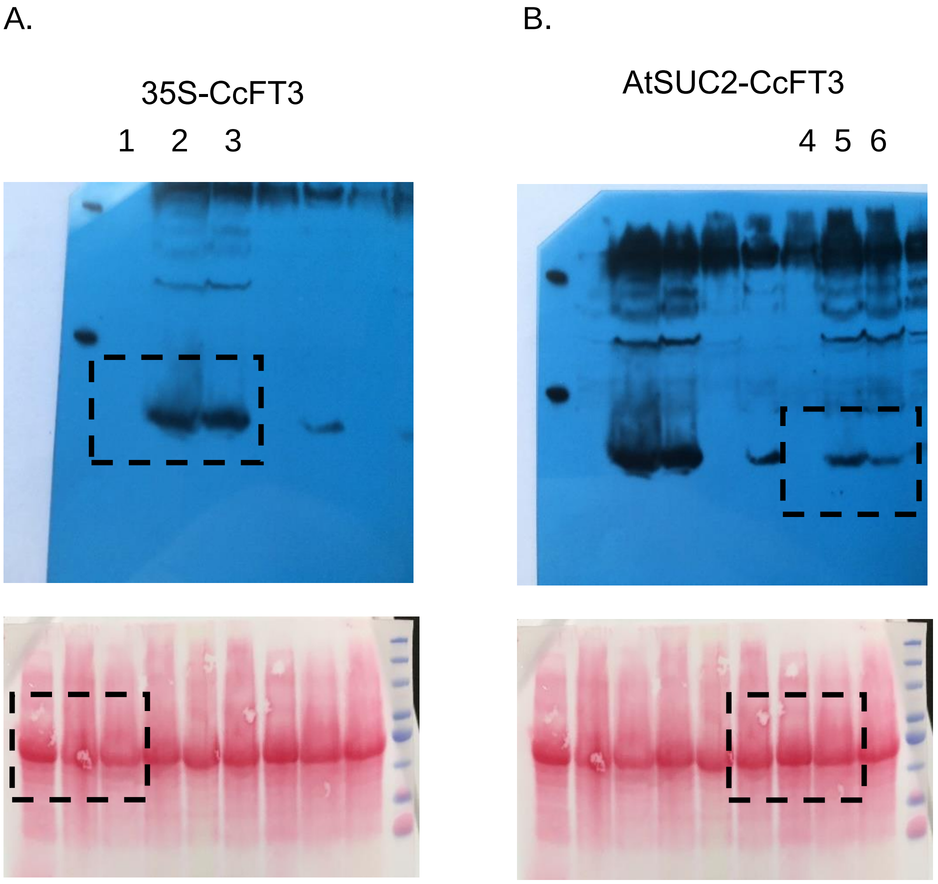

Supplementary Fig. S11. Uncropped original Western blot images of Supplementary Fig. S1 showing Arabidopsis non-transgenic and (A) 35S-CcFT3 and (B) AtSUC2-CcFT3 transgenic lines. Lanes were loaded as following: (1) Col-0 wild type non-transgenic, (2) 35S-CcFT3 seedling number 1, (3) 35S-CcFT3 seedling number 2, (4) Col-0 wild type non-transgenic, (5) AtSUC2-CcFT3 seedling number 1 and (6) AtSUC2-CcFT3 seedling number 2.
